# ﻿Contributions on a small collection of the former Subulinidae Fischer & Crosse, 1877 (Eupulmonata, Achatinoidea) with catalogue of the *Glessula* and *Rishetia* species recorded from Myanmar

**DOI:** 10.3897/zookeys.1208.116083

**Published:** 2024-07-30

**Authors:** Nem Sian Man, Jonathan D. Ablett, Ngwe Lwin, Chirasak Sutcharit, Somsak Panha

**Affiliations:** 1 Animal Systematics Research Unit, Department of Biology, Faculty of Science, Chulalongkorn University, Bangkok, 10330, Thailand Chulalongkorn University Bangkok Thailand; 2 Department of Zoology, University of Yangon, University Avenue Road, Kamayut Township 11041, Yangon, Myanmar University of Yangon Yangon Myanmar; 3 Department of Life Sciences, The Natural History Museum, Cromwell Road, London SW7 5BD, UK The Natural History Museum London United Kingdom; 4 Fauna and Flora International, No. 35, 3rd Floor, Shan Gone Condo, Myay Ni Gone Market Street, Sanchaung Township, Yangon, Myanmar Fauna and Flora International Yangon Myanmar; 5 Academy of Science, The Royal Society of Thailand, Bangkok 10300, Thailand Academy of Science, The Royal Society of Thailand Bangkok Thailand

**Keywords:** Conservation, molluscs, Southeast Asia, systematics, taxonomy, type specimen

## Abstract

The taxonomy of subulinid snails in Myanmar has been evaluated, resulting in the recognition of 40 species and subspecies across nine genera: *Allopeas*, *Bacillum*, *Curvella*, *Glessula*, *Opeas*, *Paropeas*, *Rishetia*, *Tortaxis*, and *Zootecus*. Nine species are re-described based on recently collected specimens, and two new species, *Glessulamandalayensis* Man & Panha, **sp. nov.** from Mandalay Region and *Tortaxiscylindropsis* Man & Panha, **sp. nov.** from Shan State are introduced. The genitalia and radula of *Zootecuspullus* was studied for the first time. This study also presents a comprehensive list of all subulinid species recorded to date from Myanmar. The type specimens and authenticated museum specimens have been illustrated with accompanying taxonomic remarks and nine species formerly assigned in *Glessula* are now placed in *Rishetia*: *R.akouktoungensis*, *R.baculina*, *R.basseinensis*, *R.burrailensismaxwelli*, *R.kentungensis*, *R.limborgi*, *R.nathiana*, *R.pertenuis*, and *R.pertenuismajor*.

## ﻿﻿Introduction

The Subulinidae Fischer & Crosse, 1877 is a highly diverse land snail family comprised of more than 800 species and approximately 80 genera which are native to tropical and subtropical regions in South and Central America, Africa, and South and Southeast Asia (Gude 1914; Naggs 1994; Schileyko 1999; D'ávila et al. 2020; Horsák et al. 2020). In general, subulinid snails are characterised by having a small to medium-sized (6–66 mm in height), ovate to slender, elongated conical shell, a mostly turreted spire, smooth to strong sculptures, and a concave or straight and truncated or continuous columella (Pilsbry 1906; Godwin-Austen 1920; Schileyko 1999; Do and Do 2014). The genitalia consist of a simple and slender to muscular penis, with (sometimes variously developed flagella) or without epiphallus (Gude 1914; Godwin-Austen 1920; Schileyko 1999; Budha 2017; Budha et al. 2017; D'ávila et al. 2018).

The earliest overview of molecular phylogenetic studies of the stylommatophorans revealed the ‘achatinoid’ clade, which principally comprises the Achatinoidea Swainson, 1840 [including the subulinid snails] and the Streptaxoidea Gray, 1860 (Wade et al. 2001, 2006). It confirms the traditional and widely accepted hypothesis that the Subulinidae belong within the Achatinoidea. However, the systematic classification of the achatinoideans has varied considerably and has been subject to change throughout time. The name ‘Subulininae’ was initially nominated, and then was subsequently raised to the family level under the ‘Achatinacea’ [= Achatinoidea] (Fischer and Crosse 1877; Thiele 1931: 549). This classification was later followed by Zilch (1959) and Vaught (1989). At the same time, some studies considered ‘subulinids’ as a subfamily of the Achatinidae (Inkhavilay et al. 2019; Neubert and Bochud 2020; MolluscaBase 2023). Whereas Schileyko (1999) reclassified the ‘subulinids’ as its own superfamily consisting of the SubulinidaeGlessulidae Godwin-Austen, 1920, Micractaeonidae Schileyko, 1999 and Ferrussaciidae Bourguignat, 1883, and recognised nine subfamilies within the Subulinidae. Recently, the multi-gene phylogeny of the Achatinoidea revealed the (sub)family as classified in Zilch (1959), Vaught (1989) and Schileyko (1999) are polyphyletic (Fontanilla et al. 2017). More recently, Bouchet et al. (2017) treated ‘Subulinidae sensu by earlier authors’ as a subfamily of the Achatinidae. Additionally, taxonomic placements within the Achatinoidea have also varied substantially since the revisions based on exhaustive anatomical characters had not been provided. Therefore, systematic classification of the Achatinoidea remains a source of discussion but surpasses the scope of this work, which aims to highlight the Subulinidae from Myanmar, as classified in Zilch (1959) and Vaught (1989), and to attract and encourage further systematic research.

The subulinid snails are highly diverse and primarily found in Africa and also in Southeast Asia. In Myanmar, six genera are known: *Bacillum* Theobald, 1870, *Curvella* Chaper, 1885, *Glessula* von Martens, 1860, *Opeas* Albers, 1850, *Prosopeas* Mörch, 1876, and *Zootecus* Westerlund, 1887 with a total of 37 nominal species being documented (Gude 1914; Godwin-Austen 1920). Most of these nominal species have been described based solely on shell morphology, with uncertain taxonomic status and distribution due to the scarcity of detailed illustrations of their respective type specimens and a lack of newly collected samples for over a century since the work of Gude (1914) and Godwin-Austen (1920). Therefore, this work focuses on examining the historical museum specimens, both type and non-type, and also newly collected materials, with the aim to update the taxonomic knowledge of achatinoid snails from Myanmar. Recent specimen collections were conducted in collaboration with the Forest Department of Myanmar and Fauna and Flora International (FFI), as described in our previously published taxa (Man et al. 2022, 2023). This paper will enhance the knowledge of the Subulinidae diversity in Myanmar, contribute to our understanding of land snail biogeography and will serve as a valuable resource for the improvement of the existing taxonomy of the subulinid taxa.

## ﻿﻿Materials and methods

### ﻿﻿Sampling and morphological studies

**Figure 1.**
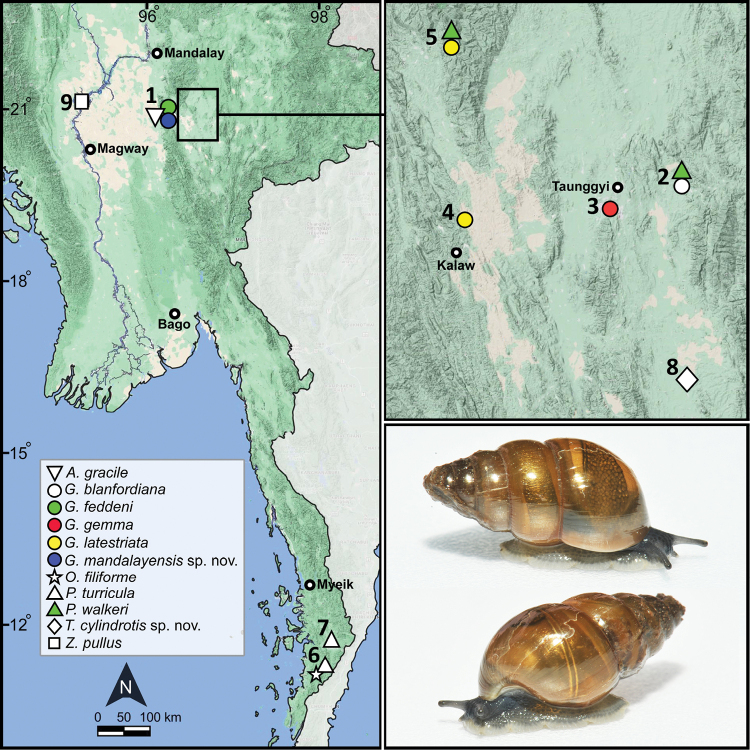
Approximate collecting localities of the subulinids from Myanmar examined in this study (right), inset figure (upper right) indicates the collecting site from Shan State, and living snails (lower right) of *Glessulamandalayensis* sp. nov., paratype CUMZ 13073 from Pyinyaung Village, Mandalay Region, Myanmar (SH ~ 14 mm). The numbers correspond to localities listed in Table 1.

Between 2015 and 2016, malacofauna surveys of the limestone habitats in Myanmar were resumed through collaborative efforts involving the Animal Systematics Research Unit (ASRU), the Forest Department of Natural Resources and Environmental Conservation and Forestry, Myanmar, and Fauna and Flora International (FFI), under the framework of an MOU (Letter No. 0092). During field trips, small samples of subulinid snails were primarily collected in Shan State and the Mandalay Region within the northeastern part of the country, as well as in Kayin and Mon states and the Tanintharyi Region in the southeast, as shown in Fig. 1.

**Figure 2.**
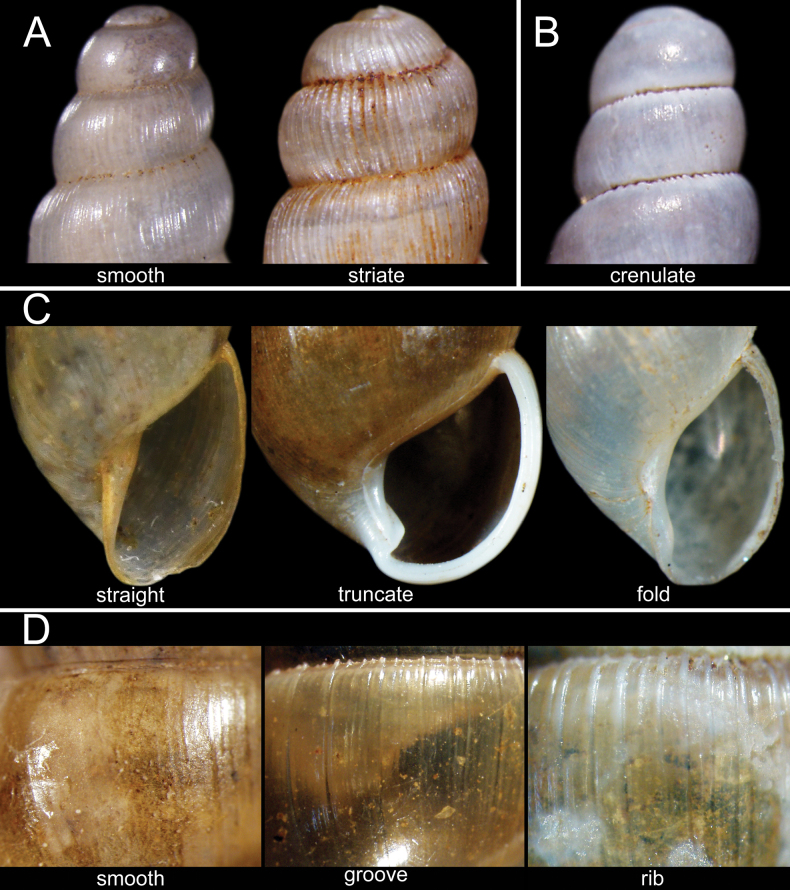
Schematic shell morphology of the subulinid snails **A** protoconch sculptures as recognised in this study: smooth and striate **B** crenulate suture **C** columella shape as recognised in this study: straight, truncate and fold **D** shell surface sculpture of the *Glessula* as recognised in this study: smooth, groove, and ribbed.

These newly collected samples were deposited in the Chulalongkorn University, Museum of Zoology (**CUMZ**), Thailand. The specimens were examined for shell morphology (Fig. 2) and reproductive system structure (when available), and the terminology used in this study follows Gude (1914), Godwin-Austen (1920), Schileyko (1999), and Budha et al. (2017). Specimens were compared to the type specimens and available authenticated collections. Adult and intact shells were measured with a digital calliper for shell height (**SH**; largest length from the apex to the base of aperture), shell width (**SW**; widest diameter from one side of the last whorl to the outermost side of aperture), and whorl counts (Kerney and Cameron 1979; Table 1).

**Table 1.** Shell measurements of subulinid species examined in this study. SH: shell height; SW: shell width. The localities numbers are indicated in Fig. 1.

Species, locality no., and CUMZ no.	No. of specimens	Ranges, mean ± S.D. in mm		SH/SW ratio	No. of whorls
		Shell height	Shell width		
*Allopeasgracile*					
1. Pyinyaung Village, Meiktila, Mandalay (13066)	8	9.8–12.1 10.76 ± 0.92	2.9–3.3 3.08 ± 0.169	3.37–3.78 3.48 ± 0.17	7½–8
*Glessulablanfordiana*					
2. Aik Kham Cave, Taunggyi, Shan (13067)	3	7.7–8.6 8.16 ± 0.47	3.7–3.9 3.81 ± 0.08	2.05–2.26 2.141 ± 0.10	6–6½
*Glessulafeddeni*					
1. Pyinyaung Village, Meiktila, Mandalay (13068)	8	7.8–10.8 9.72 ± 1.14	4.0–4.8 4.47 ± 0.33	1.95–2.25 2.16 ± 0.11	6½ –7
*Glessulagemma*					
3. Montawa Cave, Taunggyi, Shan (13069)	5	8.7–9.2 8.99 ± 0.28	3.4–3.9 3.61 ± 0.22	2.37–2.58 2.49 ± 0.09	6½–7
*Glessulalatestriata*					
4. Thale Cumon Temple, Kalaw City, Shan (13070)	4	8.4–10.8	4.0–4.7	1.86–2.31	6½–7
5. Ywangan Village, near Lin Way Monastery, Kalaw, Shan (13071)		9.22 ± 1.14	4.51 ± 0.18	2.04 ± 0.23	
*Glessulamandalayensis* sp. nov.					
1. Pyinyaung Village, Meiktila, Mandalay (13072, 13073)	30	13.3–16.0 14.32 ± 0.72	6.7–7.8 7.19 ± 0.30	1.87–2.12 1.99 ± 00.07	6–7
*Opeasfiliforme*					
6. Phra (Buddha) Cave, Tanintharyi (13074)	15	8.1–11.2 9.51 ± 1.00	2.4–3.4 2.82 ± 0.30	3.32–3.49 3.37 ± 0.05	7½–8
*Paropeasturricula*					
6. Phra (Buddha) Cave, Tanintharyi (13075)	3	11.7–15.0	3.00–3.90	3.78–3.9	8, 8½
7. Lampane Village, Tanintharyi (13076)		13.00 ± 1.72	3.4 ± 0.45	3.84 ± 0.58	
*Paropeaswalkeri*					
2. Aik Kham Cave, Taunggyi, Shan (13077)	5	8.4–15.3	3.3–3.8	2.54–4.02	8–8½
5. Ywangan Village, near Lin Way Monastery, Kalaw, Shan (13078)		12.65 ± 1.76	3.54 ± 0.22	3.54± 0.56	
*Tortaxiscylindropsis* sp. nov.					
8. Parpant area, Taunggyi, Shan (13079, 13080)	35	9.3–11.7 10.29 ± 1.06	2.1–2.5 2.27± 0.16	4.11–5.00 4.53 ± 0.39	8–9½
*Zootecuspullus*					
9. Dhammayazaka Pagoda, Bagan, Mandalay (13081, 13082)	90	11.2–13.3 11.99 ± 0.75	4.3–4.8 4.55 ± 0.16	2.48–2.80 2.63 ±0.14	8–9½

The following species list was produced using both the literature and collections, the primary type specimens (i.e., holotype, lectotype, syntype(s), and neotype) and secondary type specimens [paratype(s) and paralectotype(s)]. The taxa are arranged in alphabetical order according to their current taxonomic status. The references for the usage of each taxon name are provided herein. The name in the original combination is given with bibliographic information on the original description. The type locality is given, and if possible, the modern name and/or regional names of the type locality are provided in square brackets. Where necessary, remarks are given on the status of type specimens, authorships, availability of name, notes on the type locality, and further valuable comments.

### ﻿﻿Institutional abbreviations

**ANSP** Academy of Natural Science of Philadelphia, Drexel University, Philadelphia

**B.M.** used in old labels for the British Museum (now The Natural History Museum (NHM), London)

**CUMZ** Chulalongkorn University Museum of Zoology, Bangkok

**MNHN** Muséum National ďHistoire Naturelle, Paris

**NHMUK** The Natural History Museum, London

**SM** Forschungsinstitut und Naturmuseum Senckenberg, Frankfurt am Main

**UMZC** University Museum of Zoology Cambridge, Cambridge

**ZMB** Museum für Naturkunde, Humboldt University, Berlin

## ﻿﻿Systematic account

**Family Subulinidae Fischer & Crosse, 1877**

### ﻿﻿Genus Allopeas

Taxon classificationAnimaliaStylommatophoraSubulinidae

Baker, 1935

CA0B623A-CC7D-5A4D-A7AC-AEB89408DC97

Lamellaxis (Allopeas) Baker, 1935: 84. Zilch 1959: 349.
Allopeas —Schileyko 1999: 509.

#### Type species.

*Bulimusgracilis* Hutton, 1834, by original designation.

#### Diagnosis.

Shell slender and conical; spire high and gradually attenuated; embryonic whorls pointed and smooth; subsequent whorls with striations. Aperture vertical, broad, and oblong; columella straight, and columellar margin expanded near umbilicus. Penis long, fusiform shape at base then narrowing towards epiphallus, and flagellum absent; vagina cylindrical and narrow tube, and ~ 1/2 of penis length.

#### Remarks.

The genus is sometimes confused with *Paropeas* Pilsbry, 1906 and *Opeas* Albers, 1850. *Allopeas* can be distinguished from *Paropeas* by its less turreted shell, finner striations, smooth embryonic whorls, straight columella, and columellar margin expanded (Table 2). *Paropeas* possesses a mostly turreted shell, stronger irregular striations throughout and entire whorls, concave columella, and columellar margin less expanded. Additionally, *Allopeas* can be differentiated from *Opeas* by having larger and broader shell, attenuated spire, and stronger striations (Schileyko 1999). By contrast, *Opeas* has a smaller and narrower shell, cylindrical or less attenuated spire, and finer striations (Pilsbry 1906).

**Table 2.** Comparison of shell morphology among subulinid genera recorded in Myanmar. The superscript numbers are references; ^1^ = Pilsbry (1906), ^2^ = Gude (1914), ^3^ = Godwin-Austen (1920), ^4^ = Naggs (1994), ^5^ = Schileyko (1999), and ^6^ = Budha et al. (2017).

Genus (type species)	Shell shape	Spire	Sculpture		Aperture shape	Columella
			Embryonic whorl	Spire whorl		
***Allopeas* (*Bulimusgracilis* Hutton, 1834)^5^**	slender conical	high and gradually attenuated	smooth	striations	vertically broad; oblong	straight
***Bacillum* (*Achatinacassiaca* Reeve, 1849)^1^**	slender conical	high, turreted, and gradually attenuated	striations	obliqued striations or ribs	obliquely narrow; ovate	concave and truncated
***Curvella* (*Curvellasulcata* Chaper, 1885)^1,5^**	oblong-conical	low or high conical and rapidly attenuated	smooth	equally or irregularly spaced striations or ribs	vertically broad; ovate; oblong	straight
***Glessula* (*Achatinagemma* Reeve, 1850)^3,6^**	ovate-conical	low conical, and gradually attenuated	smooth or striations	equally spaced of striations; grooves; radial ribs	obliquely narrow or broad; ovate	concave and truncated
***Rishetia* (*Achatinatenuispira* Benson, 1836)^3,6^**	slender conical	high, turreted, and gradually attenuated	smooth or striations	equally or irregularly spaced striations or ribs	obliquely, narrow; ovate	concave and truncated
***Opeas* (*Helixgoodallii* Miller, 1822)^1,5^**	slender conical	low or high, turreted, and gradually attenuated	smooth	smooth; fine striations and growth lines	vertically narrow; oblong	concave or straight
***Paropeas* (*Bulimusacutissimus* Mousson, 1857)^1,2,4^**	slender conical	high, turreted, and gradually attenuated	striations	irregular, dense, fine or coarse striations	obliquely narrow or broad; ovate	concave or straight
***Tortaxis* (*Achatinaerecta* Benson, 1842)^1^**	cylindrical to slender conical	high, mostly turreted, narrowly or cylindrically attenuated	smooth	striations and growth lines	vertically narrow; oblong	concave or straight and with spiral fold below
***Zootecus* (*Pupainsularis* Ehrenberg, 1831)^5^**	pupiform	high, broad, and cylindrical	smooth	irregular, dense, fine or coarse striations	obliquely broad; oblong; rounded	straight

The genus *Allopeas* consists of ~ 25 species distributed across tropical regions of Asia, Europe, Africa, and America (Schileyko 1999; MolluscaBase 2023). In Southeast Asia, Vietnam has recorded four species (Schileyko 2011), while Cambodia, Laos, Myanmar, and Thailand have reported only one species, namely *Allopeasgracile* (Gude 1914; Inkhavilay et al. 2019; Sutcharit et al. 2020b).

### ﻿﻿1 Allopeas gracile

Taxon classificationAnimaliaStylommatophoraSubulinidae

(Hutton, 1834)

26FDD689-A2DC-539B-AEF1-40D497A4AAE5

Fig. 3A–D , Table 1

Bulimus (?) gracilis (?) Hutton, 1834: 84, 85, 93. Type locality: Mirzapoor, Futtehpoor Sikra, between Agra and Neemuch [Uttar Pradesh and Madhya Pradesh states, India].
Bulimus (Opeas) gracilis —Pfeiffer 1856: 156.
Stenogyra (Opeas) gracilis —von Martens 1860: 265. Pfeiffer and Clessin 1881: 321.
Spiraxis gracilis —Blanford 1861: 362.
Stenogyra gracilis —de Morgan 1885: 389, 390.
Opeas gracilis —Theobald 1878: 146. Godwin-Austen 1895: 443.
Opeas gracile —von Möllendorff 1894: 151. Pilsbry 1906: 125–132, pl. 18, figs 3–6. Gude 1914: 355–357. van Benthem Jutting 1952: 378–380.
Lamellaxis gracilis —van Benthem Jutting 1959: 131, 132.
Lamellaxis (Allopeas) gracile —Solem 1966: 94.
Allopeas gracilis —Maassen 2001: 81, 82.
Allopeas gracile —Schileyko 2011: 9. Do and Do 2014: 452, fig. 1a. Raheem et al. 2014: 117, 118, fig. 73d–f. Inkhavilay et al. 2019: 50, fig. 21a–c. Sutcharit et al. 2020b: 19.

#### Type specimens.

**Figure 3.**
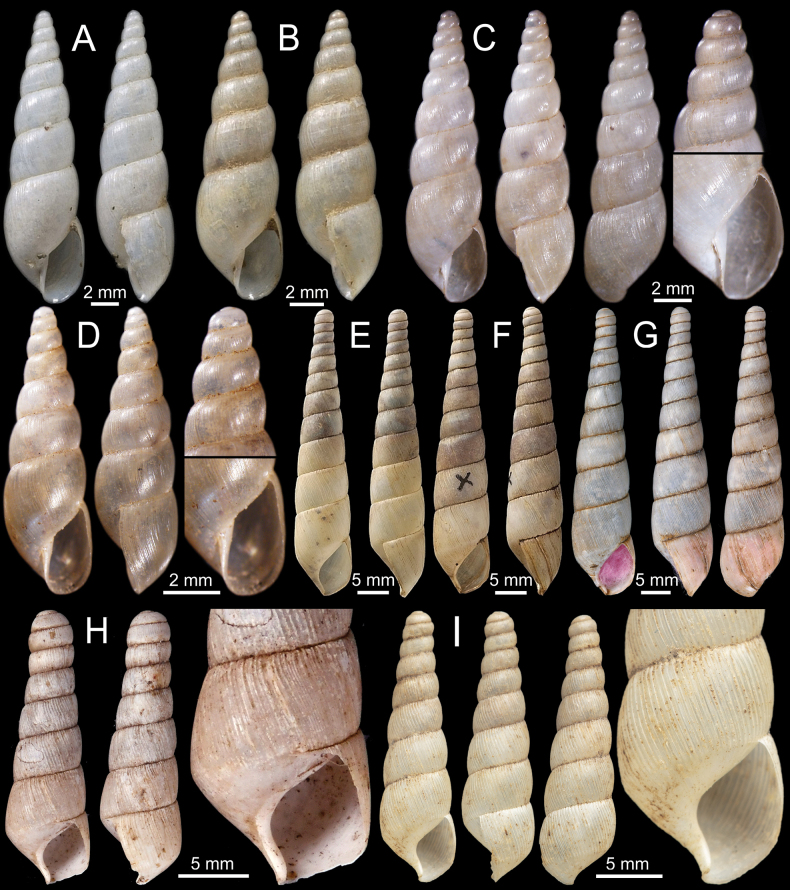
**A–D***Allopeasgracile***A** lectotype NHMUK 1856.09.15.68/1 from Mirzapore **B** paralectotypes NHMUK 1856.09.15.68/2–11 and **C, D** specimen CUMZ 13066 from Mandalay Region, Myanmar with embryonic whorls and aperture **E–H***Bacillumobtusum***E, F** syntype NHMUK 1906.2.2.349 from Bhamo, Upper Burmah **G, H** specimen NHMUK 1903.7.1.1725 from Bhamo, Upper Burma and **H** juvenile shell with last whorl **I***Bacillumtheobaldi*, syntype NHMUK 1912.4.16.121 from Burmah with last whorl.

***Lectotype***NHMUK 1856.9.15.68/1 (Fig. 3A) ex. Hutton collection from Mirzapore, designated in Raheem et al. (2014: 118). ***Paralectotypes***NHMUK 1856.9.15.68/2–11 (10 shells; Fig. 3B).

#### Other material.

Limestone hills (Apache Cement Factory), Pyinyaung Village, Meiktila District, Mandalay Region, Myanmar (20°49'39.1"N, 96°23'35.1"E): CUMZ 13066 (8 shells; Fig. 3C, D).

#### Description.

Shell conically elongated, narrow, translucent, glossy, pale yellowish colour, and with 7½–8 whorls. Apex blunt; protoconch ~ 2 whorls and smooth; subsequent whorls with obliquely more or less fine and crowded riblets. Spire gradually tapering; whorls flatly convex; suture wide and shallow; last whorl largest. Aperture high and oblong shape; peristome thin and columellar margin near umbilicus is little expanded; columella straight. Umbilicus narrowly opened.

#### Distribution.

*Allopeasgracile* is distributed worldwide in America, Africa, Europe, and Asia (Gude 1914; Horsák et al. 2020). In South Asia, it has been reported from India, Pakistan, Sri Lanka, Nepal, and all countries in Southeast Asia (van Benthem Jutting 1952; Panha 1998; Schileyko 2011; Do and Do 2014; Raheem et al. 2014; Budha et al. 2015; Foon et al. 2017; Inkhavilay et al. 2019; Nurinsiyah and Hausdorf 2019; Sutcharit et al. 2020b).

This species was previously reported in Myanmar from several localities, including Kayin State, Mon State, Kachin State, Rakhine State, Yangon Region, and Bago Region (see Gude 1914) and presently in the Mandalay Region.

#### Remarks.

Our material from the Mandalay Region matches well with the type specimen of this species. *Allopeasgracile* is a globally distributed alien species introduced to numerous countries and exhibits significant variability in both size and shape, influenced by its expansive distribution range. This species is commonly found in public parks, irrigation areas, greenhouse environments, and residential campuses (Pilsbry 1906; Neubert and Bochud 2020; Kalita 2022).

### ﻿﻿Genus Bacillum

Taxon classificationAnimaliaStylommatophoraSubulinidae

Theobald, 1870

4F3DDEDF-F4A5-5549-B06D-95361A511DE7

Achatina (Bacillum) Theobald in Hanley and Theobald 1870: 17.
Bacillum —Pilsbry 1906: 1. Gude 1914: 343. Godwin-Austen 1920: 7. Zilch 1959: 346. Schileyko 1999: 534.

#### Type species.

*Achatinacassiaca* Reeve, 1849a, subsequent designation by Pilsbry (1906: 1).

#### Diagnosis.

Shell slender and conical in shape; spire high, turreted, and gradually attenuated; embryonic whorls cylindrically rounded, and with or without radial striations; subsequent whorls with equally spaced thick or fine radial striations. Aperture oblique and narrowly ovate; columella concave and truncated, and columellar margin simple or slightly expanded.

#### Remarks.

*Bacillum* can be differentiated from *Allopeas* in being cylindrically rounded and with striations on the embryonic whorls, whilst the columella is concave and truncated (Pilsbry 1906; Schileyko 1999). While *Allopeas* has a narrowly attenuated and smooth embryonic whorls, columella straight, and columellar margin near umbilicus expanded (Table 2).

At present, this genus contains seven species mainly distributed in India, and among these, two species are known from Myanmar (Blanford 1869; Pilsbry 1906; Gude 1914; Ramakrishna et al. 2010; MolluscaBase 2023). All *Bacillum* species are known only from shell morphology, and none of the reproductive anatomy has been published so far. Godwin-Austen (1920) stated, ‘…extended knowledge of the animals of *Bacillum* and *Glessula* shows that the two genera come next each other…’, which suggests Godwin-Austen had dissected *Bacillum*, but his findings were never published.

### ﻿﻿2 Bacillum obtusum

Taxon classificationAnimaliaStylommatophoraSubulinidae

(Blanford, 1869)

EC43149C-1038-581A-A458-52A2B0794EA1

Fig. 3E–H

Achatina (Glessula) obtusa Blanford, 1869: 449. Type locality: Bhamo in regno Avae [Bhamo District, Kachin State, Myanmar].
Achatina (Bacillum) obtusa —Hanley and Theobald 1870: 17, pl. 36, fig. 6.
Achatina obtusa —Pfeiffer 1876: 290.
Glessula obtusa —Nevill 1877: 25.
Stenogyra (Subulina) obtusa —Pfeiffer and Clessin 1881: 327.
Bacillum obtusum —Pilsbry 1906: 1, 2, pl. 1, fig. 1. Gude 1914: 347.

#### Type specimens.

***Syntypes***NHMUK 1906.2.2.349 [re-registered in error as 19850143] (4 shells; Fig. 3E, F) ex. Blanford collection from Bhamo, Upper Burmah.

#### Other material.

NHMUK 1903.7.1.1725 (2 shells + 1 juvenile; Fig. 3G, H) ex. Godwin-Austen collection from Bhamo, Upper Burma. NHMUK 18/88.12.4.1012–1014 (3 shells) from Bhamo. NHMUK (1 shell) ex. TV Oldham collection from Bhamo, Burma. SMF 145947/2 (2 shells) ex. von Möllendorff collection from Bhamo.

#### Diagnosis.

Shell slender, elongate turreted and apical whorls rapidly attenuated; apex rounded, blunt and very large embryonic shell; subsequent whorls with fine radial striae. Suture shallow and whorls flattened. Aperture obliquely subovate; columella curved and truncated.

#### Distribution.

This species is known only from the type locality.

#### Remarks.

No new material of this species was collected in this study, but the syntypes are illustrated for the first time.

### ﻿﻿3 Bacillum theobaldi

Taxon classificationAnimaliaStylommatophoraSubulinidae

(Hanley, 1870)

6DDC8836-CE05-5A20-BCF6-F8F753E79F4E

Fig. 3I

Achatina (Electra) theobaldi Hanley in Hanley and Theobald 1870: 9, pl. 17, fig. 5. Type locality: Near the Salwen [near Salween River, Myanmar].
Achatina (Glessula) theobaldiana [sic]—Theobald 1870: 395.
Achatina theobaldi —Pfeiffer 1876: 290.
Stenogyra (Glessula) theobaldiana —Nevill 1878: 172.
Stenogyra (Subulina) theobaldi —Pfeiffer and Clessin 1881: 327.
Bacillum theobaldi —Pilsbry 1906: 4, pl. 1, fig. 8. Gude 1914: 344, 345. Ramakrishna et al. 2010: 150.

#### Other material.

NHMUK 1912.4.16.121 (1 shell; Fig. 3G) ex. Beddome collection from Burmah.

#### Diagnosis.

Shell elongate turreted and gradually attenuated; apex rounded, blunt and very large embryonic shell; subsequent whorls with strong and equally spaced radial ribs throughout. Aperture ovate; columella curved and truncated.

#### Distribution.

This species was recorded from near the Salween River and in Shan State, Myanmar (Hanley and Theobald 1870; Pilsbry 1906). The additional report from ‘India’ by Ramakrishna et al. (2010) is possibly erroneous.

#### Remarks.

No new material of this species was examined. *Bacillumtheobaldi* was initially proposed without a proper description other than comparing it with *B.cassiacum* (Reeve, 1849a) and with an imprecise type locality noted as ‘Near the Salwen’ (Hanley and Theobald 1870). Pilsbry (1906) confined the type locality as near to the Salween River in Shan State.

This species is superficially similar to *B.obtusum* in shell shape. However, *B.theobaldi* has equally spaced radial ridges (Fig. 3I), whereas *B.obtusum* processes finer radial striations (Fig. 3H).

### ﻿﻿Genus Curvella

Taxon classificationAnimaliaStylommatophoraSubulinidae

Chaper, 1885

DB70DB52-7EC8-534E-ABFC-A3CB63BC5EFC

Bulimus (Hapalus) Albers, 1850: 140. [non Illiger 1801 (Coleoptera)]. Type species Bulimus grateloupi Pfeiffer, 1846. Albers 1860: 238.
Curvella —Chaper, 1885: 49. Pilsbry 1906: 46. Gude 1914: 348. Zilch 1959: 351. Schileyko 1999: 515. Budha et at. 2017: 120.

#### Type species.

*Curvellasulcata* Chaper, 1885, by original designation in Chaper (1885: 48).

#### Diagnosis.

Shell oblong-conical; spire low or high and rapidly attenuated; embryonic whorls smooth; subsequent whorls with equally or irregularly spaced thick or fine radial striations or ribs. Aperture vertical, broad-ovate, or oblong, and somewhat pointed above; columella straight; columellar margin expanded near umbilicus. Penis simple and short papillate or short tube; epiphallus and flagellum absent; vagina large, muscular, and nearly equal to penis length.

#### Remarks.

*Curvella* is clearly distinct from other subulinid genera such as *Allopeas*, *Bacillum*, and *Opeas* by its oblong-conical shell, short spire, high and wide aperture, and its much broader last whorls (Table 2). Regarding the genitalia, this genus displays a more rounded, muscular, short penis and a thicker vagina than *Allopeas* and *Opeas* (Schileyko 1999). In comparison, the two latter genera have a slender, narrow, more elongated penis and vagina compared to *Curvella* (Schileyko 1999; Budha 2017). However, only genitalia of *Curvellasikkimensis* (Reeve, 1850) is known for this genus at present (Budha 2017).

This genus is widely distributed from Africa to South Asia and China, and ~ 95 species have been reported (Pilsbry 1906; Gude 1914; Schileyko 1999; Ramakrishna et al. 2010; Budha et al. 2017; MolluscaBase 2023). There are scattered reports of four *Curvella* species in Myanmar, while one species, *C.tonkiniana* Jaeckel, 1950, has been reported in Vietnam and one species, *C.jousseaumei* (de Morgan, 1885), from Peninsular Malaysia (Gude 1914; Maassen 2001; Schileyko 2011; Vermeulen et al. 2015).

### ﻿﻿4 Curvella plicifera

Taxon classificationAnimaliaStylommatophoraSubulinidae

(Blanford, 1865)

B3516C0F-BE16-54AC-B2C2-F99EE5A76B7B

Fig. 4A

Bulimus plicifer Blanford, 1865: 77. Type locality: Thayet Myo, Pegu. Pfeiffer 1868: 151. Hanley and Theobald 1874: 34, pl. 80, fig. 8.
Bulimina (Hapalus) plicifera —Pfeiffer and Clessin 1881: 300.
Buliminus (?) plicifer—Kobelt 1901: 688, 689, pl. 103, fig. 22.
Curvella plicifera —Pilsbry 1906: 63, pl. 9, fig. 45. Gude 1914: 352.

#### Type specimens.

**Figure 4.**
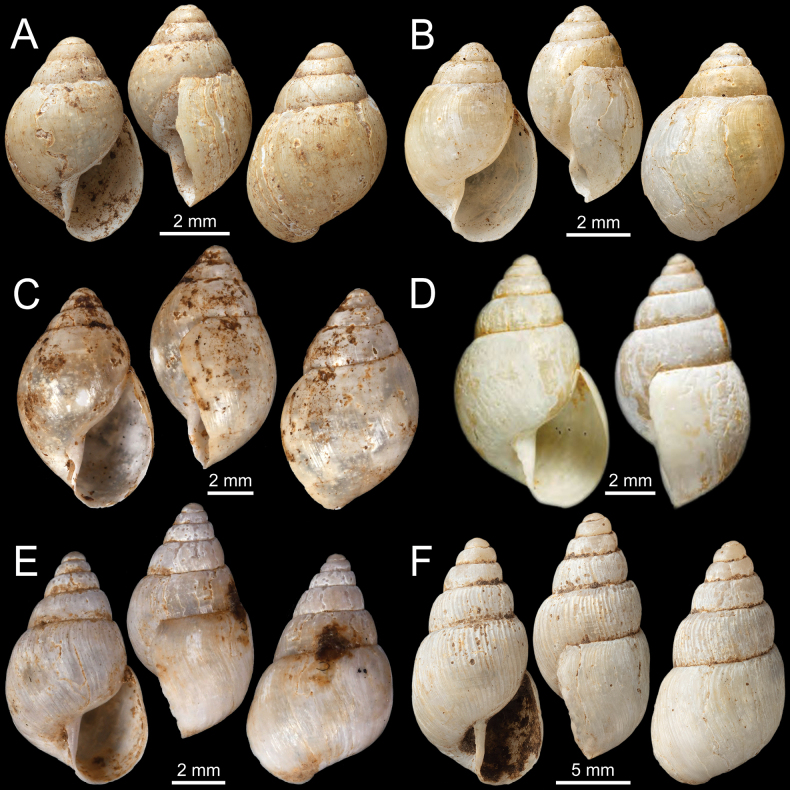
**A***Curvellaplicifera*, possible syntype NHMUK 1906.2.2.235 from Thyetmyo, Pegu; Akouktoung **B, C***Curvellapusilla***B** possible syntype NHMUK 1906.2.2.234 from Akouktong, Pegu and **C** specimen NHMUK 1888.12.4.1003–1004 from Pegu **D, E***Curvellaputa***D** holotype UMZC I.102795 from Tavoy, Birmah (after Preece et al. 2022: fig. 55f) and **E** specimen NHMUK 1888.12.4.1003–1004 from Pegu **F***Curvellascrobiculata*, possible syntype NHMUK 1906.2.2.226 from Akouktong, Pegu.

***Syntypes***NHMUK 1906.2.2.235 (2 shells; Fig. 4A) ex. Blanford collection from Thyetmyo, Pegu? Akouktoung.

#### Diagnosis.

Shell ovate conic; spire low; apex bluntly obtuse; subsequent whorls with prominent growth lines throughout. Suture shallow and whorls flattened. Aperture broadly ovate and somewhat pointed posteriorly; columella straight and expanded; parietal callus thin and with small parietal lamella. Umbilicus narrow.

#### Distribution.

This species is restricted to Myanmar and is known only from the type locality.

#### Remarks.

No new material of this species was found in this study, and only the available syntypes are examined here. The modern name of the type locality is Thayet District, Magway Region, central Myanmar [not in the Bago Region].

Among the four *Curvella* species from Myanmar, this species is similar to *C.pusilla* (Blanford, 1865) in shell shape. However, *C.plicifera* has a small parietal tooth, straight columella, and narrowly opened umbilicus, whereas *C.pusilla* possesses a smooth parietal wall (without tooth), slightly twisted columella plait, and closed umbilicus. Further confirmation from additional specimens is necessary to verify their distinctions.

### ﻿﻿5 Curvella pusilla

Taxon classificationAnimaliaStylommatophoraSubulinidae

(Blanford, 1865)

4160B10E-0536-5F24-80EE-7EB95839CB87

Fig. 4B, C

Spiraxis pusilla Blanford, 1865: 78. Type locality: Prome district, Pegu [Pyay District, Bago Region, Myanmar]. Pfeiffer 1868: 192. Hanley and Theobald 1874: 34, pl. 79, fig. 8.
Hapalus pusillus —Nevill 1878: 174.
Stenogyra [Spraxis (Euspiraxis)] pusilla—Pfeiffer and Clessin 1881: 324.
Curvella pusilla —Pilsbry 1906: 64, pl. 9, fig. 48. Gude 1914: 351.

#### Type specimens.

***Possible syntypes***NHMUK 1906.2.2.234 (2 shells; Fig. 4B) ex. Blanford collection from Akouktong, Pegu.

#### Other material.

NHMUK 1888.12.4.1003–1004 (1 shell identified as *C.pusilla*; Fig. 4C [another shell identified as *C.puta*]) ex. W. Theobald collection from Pegu.

#### Diagnosis.

Shell ovate conic; spire low; apex bluntly obtuse; subsequent whorls with prominent growth lines. Suture shallow and whorls slightly flattened. Aperture broadly ovate and pointed posteriorly; columella twisted and expanded; parietal callus thin. Umbilicus closed.

#### Distribution.

This species seems restricted to Myanmar and is known only from the type locality. The modern administrations of the type locality are ‘Prome district’ [Pyay District] and ‘Pegu’ [Bago Region].

#### Remarks.

No new material of this species was found in this study, and only the probable syntypes were examined. These are considered possible syntype material since the locality data (Akouktong, Pegu) does not precisely match that which was given in the original description (Prome district, Pegu).

Blanford (1865) noted that *C.pusilla* resembles the young specimen of *C.puta* (Benson, 1857), but this species has a closed umbilicus, while *C.puta* possesses an opened umbilicus. We recognise *C.pusilla* as a valid species following Blanford’s description, and no new materials of this species were examined in this study.

### ﻿﻿6 Curvella puta

Taxon classificationAnimaliaStylommatophoraSubulinidae

(Benson, 1857)

E9F8A342-0E56-51C2-B8D2-101CCB10AE50

Fig. 4D, E

Bulimus putus Benson, 1857: 330. Type locality: Tavoy [Dawei, Tanintharyi Region, Myanmar]. Pfeiffer 1859: 502. Blanford 1865: 94. Hanley and Theobald 1874: 34, pl. 80, fig. 9.
Hapalus putus —Nevill 1878: 178.
Bulimina (Hapalus) puta —Pfeiffer and Clessin 1881: 299.
Buliminus (?) putus—Kobelt 1901: 689, pl. 103, fig. 23.
Curvella puta —Pilsbry 1906: 63, 64, pl. 9, fig. 46. Gude 1914: 351, 352. Preece et al. 2022: 130, fig. 55f.

#### Type specimen.

***Holotype***UMZC I.102795 (Fig. 4D; after Preece et al. 2022: fig. 55f) ex. R. McAndrew ex. Benson collection from Tavoy, Birmah.

#### Other material.

NHMUK 1888.12.4.1003–1004 (1 shell identified as *C.puta*; Fig. 4E [another shell identified as *C.pusilla*]) ex. W. Theobald collection from Pegu. NHMUK 1906.1.1.1033 (1 shell) ex. Godwin-Austen collection from Bassein, Pegu.

#### Diagnosis.

Shell conical; spire high; apex obtuse; subsequent whorls with strong and prominent growth lines. Suture impressed and whorl**s** slightly convex. Aperture semi-ovate and somewhat pointed above; columella straight and dilate; parietal callus thin. Umbilicus narrow.

#### Distribution.

This species was reported from the Tanintharyi and Bago regions of Myanmar and Thailand (Blanford 1865; Gude 1914; Panha 1998).

#### Remarks.

*Curvellaputa* was described based on a single bleached specimen collected from ‘Tavoy’ in Myanmar. No new material of this species was found during this survey, but the type specimens and authenticated museum specimens are illustrated herein.

There is a mixed-species lot, NHMUK 1888.12.4.1003–1004, ex. the W. Theobald collection, labelled ‘*Hapalusputus* Benson’ from ‘Pegu’, consisting of two shells. The first shell (Fig. 4E) has a high, turreted, and pointed spire, narrowly opened umbilicus, and strong radial striations, which agree with the holotype of *C.puta* from ‘Tavoy’ (Fig. 4D). The other shell (Fig. 4C) has a lower and convex spire, fine radial striations, narrowly opened umbilicus, and without a parietal tooth, which more resembles *C.pusilla*. In comparison, *C.pusilla* possesses a closed umbilicus without a parietal tooth (Fig. 4B), while *C.plicifera* has an open umbilicus and a small parietal tooth (Fig. 4A).

This mixed-species lot suggests several interpretations: i) an extended distribution of *C.puta* beyond its type locality to the Bago Region in central Myanmar, and ii) the presence or absence of a parietal tooth and an open or closed umbilicus are possibly intraspecific variation rather than diagnostic characters distinguishing between *C.plicifera* and *C.pusilla*. Additional evidence and further specimens from a wider geographic range will clarify this issue.

### ﻿﻿7 Curvella scrobiculata

Taxon classificationAnimaliaStylommatophoraSubulinidae

(Blanford, 1865)

73AA79D4-6F3B-570C-B0CC-B98468011D7A

Fig. 4F

Bulimus scrobiculatus Blanford, 1865: 77. Type locality: Pegu, west of Irawady [Bago Region, west of Ayeyarwady River, Myanmar]. Pfeiffer 1868: 151. Hanley and Theobald 1874: 34, pl. 79, fig. 9.
Bulimina (Hapalus) scrobiculata —Pfeiffer and Clessin 1881: 300.
Hapalus scrobiculatus —Nevill 1878: 175.
Curvella scrobiculata —Pilsbry 1906: 64, 65, pl. 9, fig. 49.
Curvella scrobiculatus —Gude 1914: 350, 351.

#### Type specimens.

***Possible syntypes***NHMUK 1906.2.2.226 (2 shells; Fig. 4F) ex. Blanford collection from Akouktong, Pegu.

#### Diagnosis.

Shell conical; spire high; apex obtuse; subsequent whorls with strong and equally spaced radial ridges throughout. Suture impressed and whorls flattened convex. Aperture truncate and somewhat pointed posteriorly; columella straight and thickened; parietal callus thin. Umbilicus narrowly open.

#### Distribution.

This species is known only from the type locality and is probably an endemic species in Myanmar.

#### Remarks.

No new material of this species was found in this study, and the probably syntype is illustrated here for the first time. Blanford (1865) described three *Curvella* species, *C.plicifera*, *C.pusilla*, and *C.scrobiculata*, from Myanmar consecutively in the same publication with their type localities as ‘Pegu’ [now in the Bago Region]. Compared to the authenticated museum specimens, *C.scrobiculata* is distinct from *C.puta* by having strong radial ribs, flatter whorls, and a more turreted, pointed, and higher spire. Furthermore, *C.scrobiculata* is easily distinguishable from *C.plicifera* and *C.pusilla* by its higher, pointed, turreted spire and prominent radial ribs. In contrast, the two latter species exhibit a lower and more convex spire with finer radial striations to nearly smooth shell surfaces. These are considered possible syntype material since the locality data (Akouktong, Pegu) does not exactly match that which was given in the original description (Pegu, west of Irawady).

### ﻿﻿Genus Glessula

Taxon classificationAnimaliaStylommatophoraSubulinidae

von Martens, 1860

0C07C9B9-3D91-5570-BDBC-44A60DC4B9AA

Electra Albers, 1850: 194. [non Lamouroux 1816 (Bryozoa)]. Type species: Achatinaceylanica Pfeiffer, 1845. Adams and Adams 1855: 105. Pfeiffer 1856: 168.
Cionella (Glessula) —von Martens in Albers 1860: 254. Pfeiffer and Clessin 1881: 329.
Stenogyra (Glessula) —Nevill 1878: 166.
Glessula —Tenison-Woods 1888: 1053. Beddome 1906: 160. Pilsbry 1908: 50. Gude 1914: 377. Zilch 1959: 343. van Benthem Jutting 1952: 373, 374. Schileyko 2011: 11.

#### Type species.

*Achatinagemma* Reeve, 1850 by original designation.

#### Diagnosis.

Shell ovate-conical in shape; spire low or high conical, and regularly attenuated; embryonic whorls smooth or with striations; subsequent whorls with equally or irregularly spaced radial striations or ribs. Aperture oblique, narrow to wide, and ovate; columella concave and truncated, and columellar margin not expanded. Penis large, thick, and moderately long; epiphallus short, stout, terminally pointed, and curved; flagellum present (rarely absent) with comb-like or hand-like structures; epiphallic caecum absent; vagina muscularly and nearly equal to ~ 1/5–2/5 of penis length.

#### Remarks.

Superficially, *Glessula* and *Rishetia* are similar in having a concave and truncated columella, but they differ in their shell shape and genital structure (Godwin-Austen 1920; Raheem et al. 2014; Budha et al. 2017). In terms of shell morphology, *Glessula* can be differentiated by its ovate-conical shape, short spire, large and blunt embryonic whorls, while *Rishetia* possesses a slender, conical-shaped elongate spire which narrow and rather acute embryonic whorls (Table 2). Regarding the genital characters, *Rishetia* exhibits a simple, tubular-shaped flagellum with the epiphallic caecum present (Table 3), while *Glessula* displays a well-developed comb-like flagellum (appendage) and the epiphallic caecum absent (Budha et al. 2017); except *G.mandalayensis* sp. nov. which has no flagellum. Likewise, *Glessula* is also distinct from *Curvella* by its concave and truncated columella, not expanded columellar margin, and obliquely narrow and ovate aperture, while *Curvella* possesses a straight columella, expanded columellar margin, and more vertical, higher, and broader oblong aperture.

**Table 3.** Comparison of terminal part of male genitalia between *Glessula* and *Rishetia*. References: 1 = Semper (1874); 2 = Godwin-Austen (1918); 3 = Godwin-Austen (1920); 4 = Schileyko and Kuznetsov (1996); 5 = Schileyko (1999); 6 = Budha et al. (2017). n/a = data not available (not mentioned in the description or could not differentiated from the original figure).

Species	Epiphallic caecum	Flagellum	References
***Glessula* von Martens, 1860**			
*G.hebetata* Godwin-Austen, 1920	absent	comb-like with numerous notches	6
*G.inornata* (Pfeiffer, 1853)	absent	comb-like with numerous notches	3
*G.oakesi* Godwin-Austen, 1918	absent	comb-like with three notches	2, 3
*G.ochracea* Godwin-Austen, 1918	absent	comb-like with numerous notches	2, 3
*G.orobia* (Benson, 1860)	absent	comb-like with five notches	3, 6
*G.orophila* (Reeve, 1849)	absent	comb-like with numerous notches	1
*G.serena* (Benson, 1860)	absent	comb-like with numerous notches	5
*G.tamakoshi* Budha & Backeljau, 2017	absent	comb-like with numerous notches	6
*G.mandalayensis* sp. nov.	absent	absent	This study
***Rishetia* Godwin-Austen, 1920**			
*R.garoense* (Godwin-Austen, 1920)	n/a	long cylindrical	3
*R.hastula* (Benson, 1860)	short knob-like	short tubular	6
*R.kathmandica* Budha & Backeljau, 2017	short cylindrical	long cylindrical	6
*R.longispira* (Godwin-Austen, 1920)	n/a	long cylindrical	3, 6
*R.mastersi* (Godwin-Austen, 1920)	short cylindrical	long cylindrical	6
*R.nagarjunensis* Budha & Naggs, 2017	short knob	short knob	6
*R.rishikeshi* Budha & Naggs, 2017	short cylindrical	long cylindrical	6
*R.subulata* Budha & Naggs, 2017	short cylindrical	long cylindrical	6
*R.tribhuvana* Budha, 2017	short cylindrical	long cylindrical	6
*R.tenuispira* (Benson, 1936)	short cylindrical	long cylindrical	4, 5

*Glessula* is widely distributed throughout the Indian subcontinent, and nearly 100 species have been described (Godwin-Austen 1920; Schileyko 1999; Ramakrishna et al. 2010; MolluscaBase 2023). Among the sporadic species recorded in Southeast Asia, Myanmar has 12 species (Gude 1914; Godwin-Austen 1920), while two species, *G.latestriata* von Möllendorff, 1899 and *G.paviei*Morlet 1893, have been recorded from Vietnam, Laos, and Thailand, and another two species, *G.sumatrana* (von Martens, 1864) and *G.wallacei* (Pfeiffer, 1856) from Indonesia (Tenison-Woods 1888; van Benthem Jutting 1952; Solem 1966; Schileyko 2011; Inkhavilay et al. 2019).

In this study, only five species including a new species, *G.blanfordiana* Nevill, 1877, *G.feddeni* Godwin-Austen, 1920, *G.gemma* (Reeve, 1850), *G.latestriata*, and *G.mandalayensis* sp. nov. have been collected and re-described.

### ﻿﻿8 Glessula blanfordiana

Taxon classificationAnimaliaStylommatophoraSubulinidae

Nevill, 1877

1FAF6C14-5BA4-5EB1-A10D-36DCFF31DAF9

Fig. 5A–D , Table 1

Glessula blanfordiana Nevill, 1877: 26. Type locality: Ponsee, Yunnan [Ponsee, Kahkyen Hills, Yunnan Province, China]. Beddome 1906: 171. Pilsbry 1909: 98, pl. 13, fig. 11. Gude 1914: 437, 438. Godwin-Austen 1920: 56, pl. 164, fig. 21.
Stenogyra (Glessula) blanfordiana —Nevill 1881: 138, pl. 5, fig. 12.

#### Type specimens.

**Figure 5.**
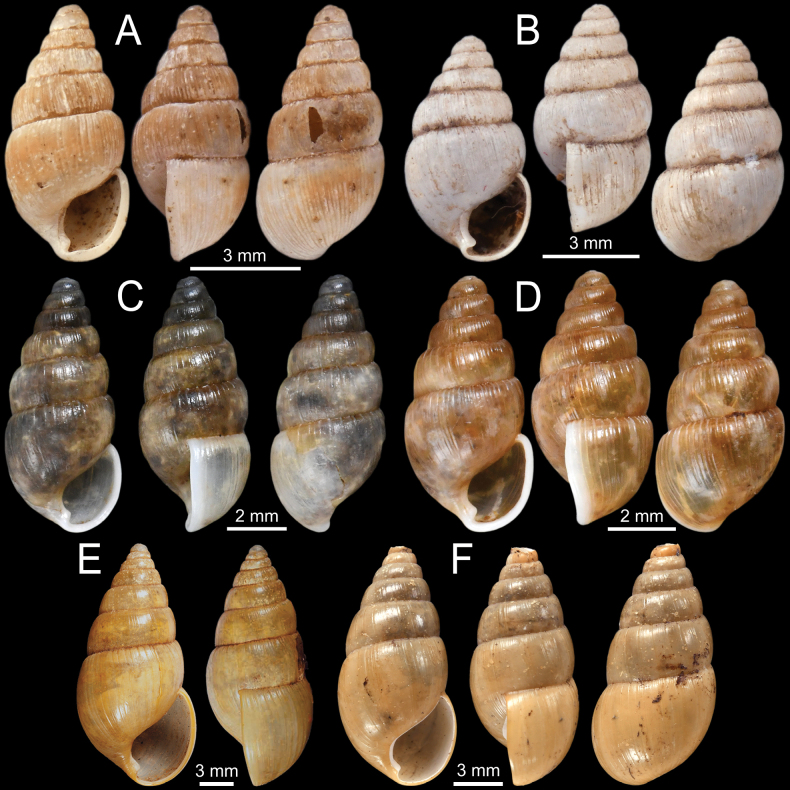
**A–D***Glessulablanfordiana***A, B** syntypes NHMUK 1988150 from Ponsee, Yunnan, W. China and **C, D** specimen CUMZ 13067 from Taunggyi, Shan State, Myanmar **E, F***Glessulacrassilabris***E** syntype UMZC I.102430 from Bengal (after Preece et al. 2022: fig. 51e) and **F** specimen NHMUK from Cherrapoonji (M10604/2).

***Syntypes***NHMUK 1988150 (2 shells; Fig. 5A, B) ex. Godwin-Austen collection from Ponsee, Yunnan, W. China.

#### Other material.

Aik Kham Cave, Taunggyi Township, Taunggyi District, Shan State, Myanmar (20°49'07.0"N, 97°13'42.0"E): CUMZ 13067 (3 shells; Fig. 5C, D).

#### Description.

Shell ovately conical, solid, brownish colour, and with 6–6½ whorls. Apex rounded; protoconch ~ 2 whorls with weak radial striations; subsequent whorls with equally and widely spaced ribs, more raised, and coarser near suture of last whorl. Spire regularly attenuated, slightly turreted; whorls convex; suture deep; last whorl largest. Aperture obliquely and narrowly ovate; peristome white and thickened; columella short, concave, slightly twisted and abruptly truncated. Umbilicus closed.

#### Distribution.

This species was originally described from Yunnan, China, and subsequently reported from Bhamo, Kachin State, Myanmar (Nevill 1881). In this study, new material has been collected from Shan State, which borders Yunnan to the east and Bhamo to the north.

#### Remarks.

When nominating this species, Nevill (1877: 26) compared it with *G.peguensis* (Blanford, 1865) from Bago and stated that *G.blanfordiana* has slender (less convex) whorls, wavy or curving, and stronger radial ribs. Nevertheless, the type specimens of these two species, *G.blanfordiana* (height 8–9 mm, Fig. 5A, B) and *G.peguensis* (height ~ 7 mm, Fig. 10B, C), exhibit considerable similarity in terms of shell shape and shell size. Therefore, more specimens from the Bago Region are necessary to ascertain whether the difference in shell sculptures represents variations or are taxonomically informative characters separating these two species.

*Glessulablanfordiana* examined herein are noticeably distinct from their congeners recorded in Shan State and Mandalay Region, namely *G.feddeni* Godwin-Austen, 1920 and *G.latestriata* von Möllendorff, 1899. Specifically, *G.blanfordiana* has a shorter and bluntly attenuated spire, more bulging whorls, deeper suture, and stronger and densely radial ribbed sculptures. In contrast, the other species exhibit higher and pointed spires, more flattened whorls, and smooth to radial grooves sculptures (see Fig. 2D).

*Glessulablanfordiana* was described based on two specimens collected by J. Anderson during the Expedition to Yunnan and Upper Burma. The original description was very brief, without shell dimensions and illustrations. However, under the note of *G.ponsiensis* Godwin-Austen, 1920, states, ‘The type from Ponsee, Yunnan (Plate CLXIV. fig. 20, apex) [= *G.blanfordiana*], has been sent me from Calcutta by the Director of the Zoological Survey of India, Dr. N. Annandale…’ (Godwin-Austen 1920: 56). The NHMUK type collections contain a single lot with an original label stating ‘Type’ from ‘Dr. J. Anderson’, and collection locality from ‘Ponsee’. These two specimens match well with the species description; both are figured herein for the first time. It is possible that N. Annandale may have gifted this type specimen lot to Godwin-Austen for comparison with other species, as was the case with other specimens from the Zoological Survey of India.

### ﻿﻿9 Glessula crassilabris

Taxon classificationAnimaliaStylommatophoraSubulinidae

(Benson, 1836)

C45F58F5-D764-559C-8764-78A469717FC3

Fig. 5E, F

Achatina crassilabris Benson, 1836: 353. Type locality: N.E. Frontier of Bengal. Pfeiffer 1848: 261. Reeve 1850: Achatina, pl. 21, fig. 81. Pfeiffer 1853: 493. Pfeiffer 1860: 313, pl. 25, figs 12, 13. Benson 1860: 464. Blanford 1865: 95.
Oleacina (Electra) crassilabris —Adams and Adams 1855: 105.
Achatina (Electra) crassilabris —Pfeiffer 1856: 168. Hanley and Theobald 1870: 17, pl. 36, fig. 1.
Cionella (Glessula) crassilabris —von Martens 1860: 254.
Stenogyra (Glessula) crassilabris—Nevill 1878: 170.
Glessula crassilabris —Godwin-Austen 1876: 315. Pfeiffer and Clessin 1881: 330. Beddome 1906: 169. Pilsbry 1909: 96, pl. 10, figs 14, 16. Gude 1914: 426. Godwin-Austen 1920: 39, 40, pl. 160, figs 14, 17–20, pl. 164, figs 16, 17. Ramakrishna et al. 2010: 157. Preece et al. 2022: 122, fig. 51e.

#### Type specimen.

***Syntype***UMZC I.102430 (1 shell; Fig. 5E after Preece et al. 2022: fig. 51e) from Bengal.

#### Other material.

NHMUK (1 shell; Fig. 5F) from Cherrapoonji (M10604/2). NHMUK 20200349 (2 shells) from N.E. Bengal. NHMUK 20200350 (2 shells) M.10695/2 from Khasi.

#### Diagnosis.

Shell oblong ovate and regularly attenuated; spire high conical; apex rounded; subsequent whorls with weak and irregularly spaced radial ridges, slightly stronger near suture, and fine radial groves present. Suture impressed and whorls slightly convex. Aperture ovate; columella strong, concave, and truncated.

#### Distribution.

This species is known from India and Rakhine State, Myanmar (Godwin-Austen 1920).

#### Remarks.

The taxonomic history and clarification status of the type specimens have recently been published in Preece et al. (2022). Blanford (1865: 95) reported a small variety of *Glessulacrassilabris* based on specimens from ‘Arakan’ [Rakhine State] and ‘Shan hills near Ava’ [Shan Hills near Innwa], and also suggested that the latter specimen lot were possibly distinct from *G.crassilabris* s. s., although they were closely related. The following year, Godwin-Austen (1920) introduced the Shan hills specimens as *G.feddeni* Godwin-Austen, 1920, but the small variety from ‘Arakan’ has never been made available.

### ﻿﻿10 Glessula feddeni

Taxon classificationAnimaliaStylommatophoraSubulinidae

Godwin-Austen, 1920

6E266811-260D-5961-9498-F61E9264945F

Fig. 6 , Table 1

Glessula feddeni Godwin-Austen, 1920: 58, pl. 162, fig. 15. Type locality: Shan Hills [Shan State, Myanmar].
Glessula feddeni var.—Godwin-Austen 1920: 58, pl. 163, fig. 14.

#### Type specimens.

**Figure 6.**
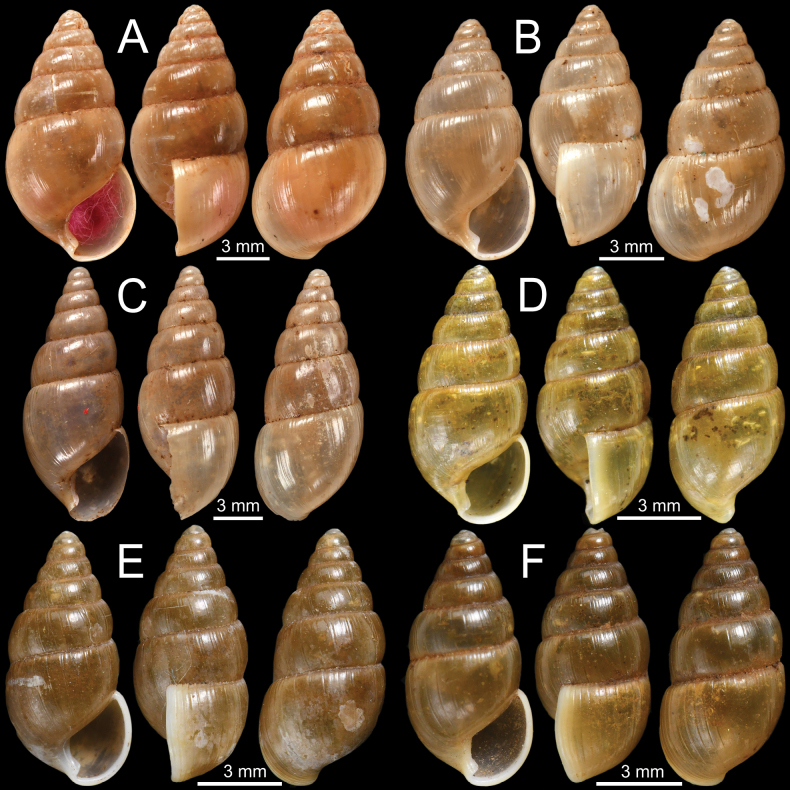
**A–F***Glessulafeddeni***A, B** syntypes NHMUK 1906.2.2.261 from Shan Hills, E. of Ava **C** specimen NHMUK 1906.3.3.41 from Shan Hills and **D–F** specimen CUMZ 13068 from Pyinyaung Village, Mandalay Region, Myanmar.

***Syntypes***NHMUK 1906.2.2.261 [re-registered in error as 1985142] (5 shells; Fig. 6A, B) ex. Blanford ex. Godwin-Austen collection from the Shan Hills, E. of Ava.

#### Other material.

NHMUK 1906.3.3.41 (1 shell; Fig. 6C) ex. Blanford ex. Fedden collection labelled as ‘var.’ from the Shan Hills. Limestone hills (Apache Cement Factory), Pyinyaung Village, Meiktila District, Mandalay Region, Myanmar (20°49'39.1"N, 96°23'35.1"E): CUMZ 13068 (8 shells; Fig. 6D–F).

#### Description.

Shell globosely conical, solid, glossy, brownish colour, and with 6½–7 whorls. Apex slightly pointed; protoconch ~ 2 whorls with smooth surface; subsequent whorls with strong, oblique, and equally spaced radial grooves that more distinct near suture. Spire attenuated, turreted and pointed; whorls flatly convex; suture deep; last two whorls almost equal and largest. Aperture narrowly and obliquely ovate; parietal wall straight; peristome white and thickened; columella short, concave, slightly twisted, and truncated. Umbilicus closed.

#### Distribution.

This species was originally described from ‘Shan Hills’, but the precise type locality was not provided. In this study, *G.feddeni* was located in the Mandalay Region adjacent to the Shan Hills.

#### Remarks.

Godwin-Austen (1920) clearly states the catalogue number ‘Type No. 261.06.2.2’ (NHMUK 1906.2.2.261) from the Shan Hills. The original description included an illustration and two sets of measurements. The Godwin-Austen’s type lot in the NHMUK consists of five shells with an original handwritten label stating ‘Type’. The two specimens with the largest shell height closest to the given measurements are here illustrated together with another shell from the syntype lot.

Godwin-Austen (1920: 58, pl. 163, fig. 14) also recognised another variety as ‘var.’ and illustrated specimen lot ‘No. 41.06.3.3’ (NHMUK 1906.3.3.41). Nevertheless, this entity has never been properly made available. The specimen lot NHMUK 1906.3.3.41 (Fig. 6C) labelled as ‘var.’ is excluded from the type series of this nominal species (ICZN 1999: Art. 72.4.1). However, this specimen has a more slender shell than the type series (Fig. 6A, B) and is possibly more closely related to *G.latestriata*, which is also distributed in the Shan State.

### ﻿﻿11 Glessula gemma

Taxon classificationAnimaliaStylommatophoraSubulinidae

(Reeve, 1850)

564D9BB6-9C40-5DD7-A49E-44B00E351BD9

Fig. 7A–D , Table 1

Achatina gemma Reeve, 1850: pl. 22, fig. 123 (Benson MSS). Type locality: Barrackpore, Bengal. Pfeiffer 1853: 496. Benson 1860: 464.
Oleacina (Electra) gemma —Adams and Adams 1855: 105.
Achatina (Electra) gemma —Pfeiffer 1856: 168. Hanley and Theobald 1870: 17, pl. 36, fig. 7.
Cionella (Glessula) gemma —von Martens 1860: 254.
Stenogyra (Glessula) gemma —Nevill 1878: 170.
Glessula gemma —Pfeiffer and Clessin 1881: 331. Beddome 1906: 169. Pilsbry 1909: 97, pl. 13, figs 1, 3. Gude 1914: 428. Godwin-Austen 1920: 22. Raheem et al. 2014: 124, figs 78b, 79c, d. Preece et al. 2022: 235, 236.

#### Type specimen.

The type specimen could not be located in the UMZC nor in the NHMUK collections (Raheem et al. 2014; Preece et al. 2022).

#### Other material.

**Figure 7.**
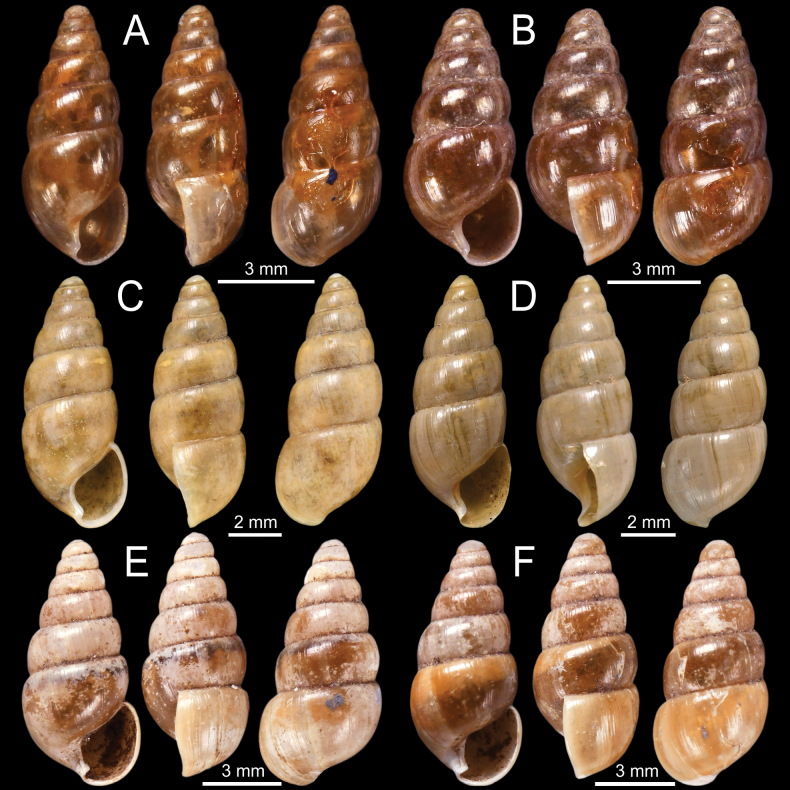
**A–D***Glessulagemma***A, B** specimen NHMUK 1946.10.16.45–53 from Bengal and **C, D** specimen CUMZ 13069 from Taunggyi, Shan State, Myanmar **E, F***Glessulainedita*, syntypes NHMUK 1906.5.5.88 from Shan Hills.

NHMUK 1946.10.16.45–53 (9 shells; Fig. 7A, B) ex. Benson collection from Bengal. Montawa Cave, Taunggyi City, Taunggyi District, Shan State, Myanmar. (20°45'15.9"N, 97°1'3.4"E): CUMZ 13069 (5 shells; Fig. 7C, D).

#### Description.

Shell elongate ovate, solid, purple-black or yellowish colour, and with 6½–7 whorls. Apex blunt; protoconch ~ 2 whorls with smooth surface; subsequent whorls with smooth and glossy to weakly radial ribs. Spire grows evenly, is high and obtuse; whorls flatly convex; suture wide and shallow; last two whorls almost equal and largest. Aperture obliquely ovate; peristome moderately thickened and white; columella short, concave, and truncated. Umbilicus closed.

#### Distribution.

*Glessulagemma* was originally described from India and then its range extended to Bangladesh (Gude 1914; Godwin-Austen 1920; Raheem et al. 2014). In Myanmar, this species has been recorded from the Rakhine State and now in the Shan State.

#### Remarks.

The historical specimens NHMUK 1946.10.16.45–53 ex. Benson collection from Bengal is illustrated herein as an example.

Our specimens match well with the authenticated Reeve specimen of this species, although it is far from the type locality and previously reported region in the Rakhine State, Myanmar. Among all the *Glessula* species from Myanmar, *G.gemma* is superficially similar to *G.latestriata* from Shan State. In comparison, *G.gemma* has smooth shell surfaces, less turreted spire, and a shallow suture, while *G.latestriata* possesses well-developed grooves, a turreted and more attenuated spire, and a deeper suture.

### ﻿﻿12 Glessula inedita

Taxon classificationAnimaliaStylommatophoraSubulinidae

Godwin-Austen, 1920

B8348A9C-4069-519F-B612-F77FC53E0821

Fig. 7E, F

Glessula ineditus Godwin-Austen, 1920: 58, 59. Type locality: Shan Hills [Shan State, Myanmar].

#### Type specimens.

***Syntypes***NHMUK 1906.5.5.88 [re-registered in error as 1985228] (3 shells; Fig. 7E, F) ex. W.T. Blanford collection from Shan Hills.

#### Diagnosis.

Shell oblong turreted and regularly attenuated; spire high conical; apex rounded; subsequent whorls with very weak irregularly spaced radial ribs. Suture deep and whorls convex. Aperture widely ovate; columella concave and truncated.

#### Distribution.

This species is known to occur only from the type locality.

#### Remarks.

The original description did not include an illustration, and one set of shell measurements was given. Godwin-Austen (1920: 58) clearly states, ‘Type No. 88.06.5.5’ (NHMUK 1906.5.5.88) is from the Blanford collection. This syntype lot consists of three shells, and the original label states ‘Type’. The largest specimen matches well with the given measurements, and another shell from the same syntype lot is illustrated herein for the first time. No new materials have been reported to date.

### ﻿﻿13 Glessula latestriata

Taxon classificationAnimaliaStylommatophoraSubulinidae

von Möllendorff, 1899

6689D622-9415-5ACF-8A32-4787D4A53B34

Fig. 8A–D , Table 1

Glessula latestriata von Möllendorff, 1899: 166. Type locality: Kalow, Shan State [Kalaw Township, Taunggyi District, Shan State, Myanmar]. Beddome 1906: 172. Pilsbry 1909: 100. Gude 1914: 443. Godwin-Austen 1920: 59. Solem 1966: 93, 94, pl. 2, fig. e. Zilch 1973: 110, pl. 5, fig. 26. Inkhavilay et al. 2019: 49, fig. 20c, d.

#### Type specimens.

**Figure 8.**
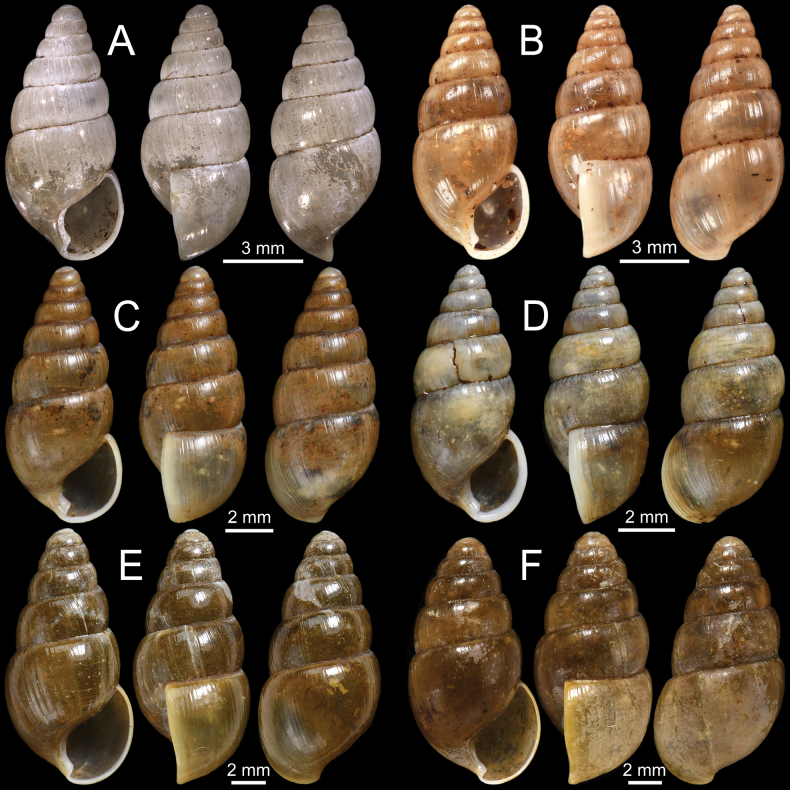
**A–D***Glessulalatestriata***A** lectotype SMF 145919 from Kalow, Shan States **B** paralectotypes NHMUK 1926.2.3.19–20 from Kalow, Shan States **C** specimen CUMZ 13070 from Thale Cumon Temple, Kalaw, Shan State, Myanmar and **D** specimen CUMZ 13071 from Ywangan Village, Kalaw, Shan State, Myanmar **E, F***Glessulamandalayensis* sp. nov. **E** holotype CUMZ 13072 from Pyinyaung Village, Mandalay Region, Myanmar and **F** paratypes CUMZ 13073 from the type locality. Photograph: Gojšina & Páll-Gergely (**A**).

***Lectotype*** SMF 145919/1 (Fig. 8A) designated in Zilch (1973: pl. 5, fig. 26) from Kalow, Shan States. ***Paralectotypes*** SMF 227513/2 (2 shells) and NHMUK 1926.2.3.19–20 (2 shells; Fig. 8B) from Kalow, Shan States.

#### Other material.

Thale Cumon Temple, Kalaw City, Shan State, Myanmar (20°43'24.1"N, 96°35'38.9"E): CUMZ 13070 (3 shells; Fig. 8C). Ywangan Village, near Lin Way Monastery, Kalaw Township, Taunggyi District, Shan State, Myanmar (21°13'43.3"N, 96°33'19.2"E): CUMZ 13071 (1 shell; Fig. 8D).

#### Description.

Shell ovate conic, solid, glossy, brownish colour, and with 6½–7 whorls. Apex blunt; protoconch ~ 2 whorls with smooth to shallow radial grooves; subsequent whorls with distinctly thickened and equally spaced grooves. Spire gradually attenuated, slightly turreted, and bluntly pointed; whorls convex; suture deep; last two whorls almost equal and largest. Aperture narrow and obliquely ovate; peristome white and thickened; columella short, concave, slightly twisted, and truncated. Umbilicus closed.

#### Distribution.

This species is known from Shan State in Myanmar, northern Thailand, and Laos (Gude 1914; Solem 1966; Inkhavilay et al. 2019).

#### Remarks.

von Möllendorff (1899) described this species based on specimens collected by B Strubell from Shan State, Myanmar. The original description did not include any illustrations, and only one set of measurements was given. The species description was not explicitly based on one specimen. Nevertheless, Zilch (1973: 110) later used the terms ‘Holotypus SMF 145919’ and ‘Paratypen SMF 227513/2’ for the von Möllendorff type series in the SMF collection. This is not a valid holotype designation, but we consider it an inadvertent lectotype designation (ICZN 1999: Arts. 73.1, 74.5, and Recommendation 73F). The other specimens labelled as ‘Paratypen SMF 227513/2’ and ‘Co-types NHMUK 1926.2.3.19–20’ are considered paralectotypes.

Although both *G.latestriata* and *G.feddeni* possess radial grooves striations, the former has a slender shell, gradually attenuated spire, and blunter apex; in contrast, *G.feddeni* has a larger and broader shell, turreted, intermediately attenuated spire and pointed apex with abruptly truncate columella.

### ﻿﻿14 Glessula mandalayensis

Taxon classificationAnimaliaStylommatophoraSubulinidae

Man & Panha sp. nov.

59E60213-F640-51FF-B445-5D97969F7680

https://zoobank.org/01347449-D63E-469B-826C-BEE5D0D56B8E

**Figure 9.**
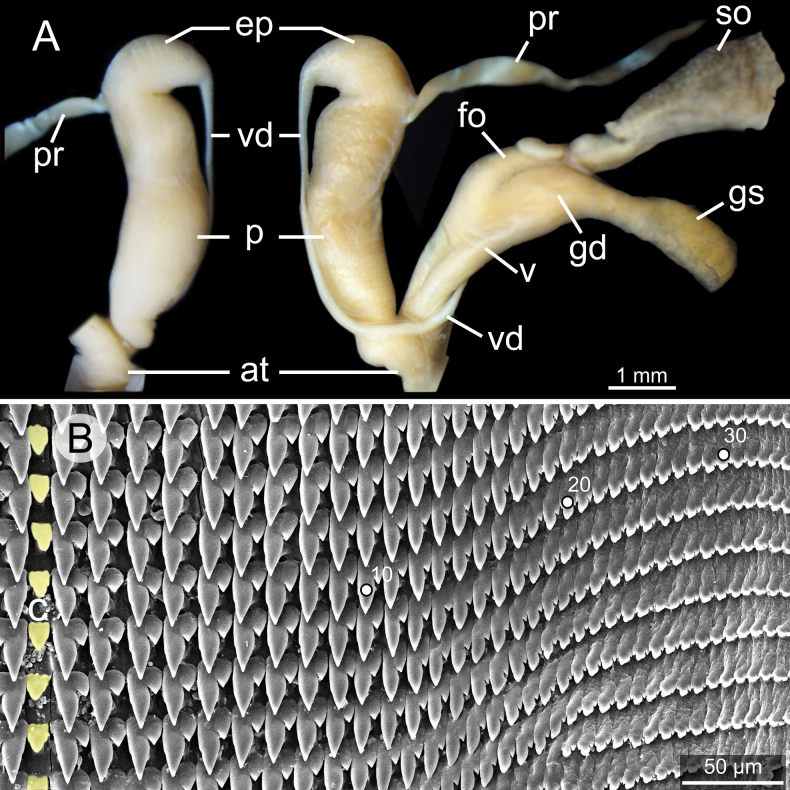
Genitalia and radula of *Glessulamandalayensis* sp. nov., paratype CUMZ 13073 from Pyinyaung Village, Mandalay Region, Myanmar **A** reproductive anatomy and **B** radula morphology: yellow colour and ‘C’ indicates central teeth row and numbers indicate tooth order from lateral to marginal end. Abbreviations: **at**, atrium; **ep**, epiphallus; **fo**, free oviduct; **gd**, gametolytic duct; **gs**, gametolytic sac; **p**, penis; **pr**, penial retractor muscle; **so**, spermoviduct; **v**, vagina; **vd**, vas deferens.

Figs 8E, F , 9 , Tables 1 , 3

#### Type specimens.

***Holotype***CUMZ 13072 (height 14.6 mm, width 7.1 mm; Fig. 8E), ***paratypes***CUMZ 13073 (29 shells; Fig. 8F), NHMUK 20230922 (2 shells) and SMF (2 shells).

#### Type locality.

Limestone Hills (Apache Cement Factory), Pyinyaung Village, Meiktila District, Mandalay Region, Myanmar (20°49'39.1"N, 96°23'35.1"E).

#### Etymology.

The specific name *mandalayensis* is the name of region, where the type specimens of this species were collected.

#### Diagnosis.

Shell large, globosely ovate; spire broad, almost cylindrically attenuated, turreted; last whorl largest; shell surface glossy, smooth to shallow grooves; aperture ovately rounded and broad; peristome thin and white.

#### Description.

Shell globosely ovate, solid, glossy, brown to bright ochraceous colour, and with 6–7 whorls. Apex blunt and large; protoconch ~ 2 whorls with smooth surface; subsequent whorls with smooth to fine radial striations or sometimes with shallow grooves. Spire broad, cylindrically attenuated, turreted; whorls flatly convex; suture deep; last whorl largest. Aperture ovately rounded and broad; peristome white and thin; columella short, concave, and truncated. Umbilicus closed.

***Genitalia*** (*n* = 1). Atrium very short. Penis evenly broad, stout, and thick muscularly. Epiphallus short, stout, slightly curved triangular shape, thick muscularly, smooth surface, and ~ 1/4 of penis length; flagellum absent. Penial retractor muscle long, thickened and attaches laterally at junction of penis and epiphallus. Vas deferens long distinct tube connected between tip of epiphallus and free oviduct and held in position to penis and vagina with weak connective tissue (Fig. 9A).

Vagina muscularly and nearly as long as penis. Gametolytic duct short and enlarged at base; gametolytic sac small and elliptical in shape. Free oviduct short and stout, and spermoviduct enlarged.

***Radula*.** Each row contains ~ 70+ teeth with half-row formula: central-lateral-marginal teeth (1–(19–20)–(15–16+)). Central tooth relatively small, symmetrical tricuspid with pointed central cusp and very small lateral cusps. Lateral teeth bicuspid: endocone long, slender and with pointed tip; ectocone small, pointed tip and located at base of teeth. Latero-marginal teeth gradually reduced in size and asymmetrically bicuspid: endocone short with pointed tip; ectocone small triangle on shape and situated around middle of tooth height. Outermost teeth small and becoming tricuspid; mesocone short and blunt tip; endocone and ectocone very small and pointed tips (Fig. 9B).

#### Distribution.

This new species is known only from the type locality.

#### Differential diagnosis.

*Glessulamandalayensis* sp. nov. can be distinguished from other congeners in Myanmar by having the largest and most globosely ovate shell, obtuse and broad spire, wide aperture, and thinner peristome. In particular, this new species differs from a sympatric species, *G.feddeni*, by having a larger size (height 13.3–16.0 mm; Table 1), more rounded shell, obtuse and broader turreted spire, wider aperture, thinner peristome, and smoother shell surface. In comparison, *G.feddeni* is smaller in size (height 7.8–10.8 mm; Table 1), with a more solid, slender conical shell, pointed and narrow spire, smaller aperture, thicker peristome, and with distinct grooves on the shell surface. *Glessulamandalayensis* sp. nov. can also be separated from *G.latestriata* by its broader shell, wider spire, and blunt apex with smoother shell surface. Whereas *G.latestriata* has a slenderer shell, narrowly attenuated spire and pointed apex with distinct radial grooves striations. This new species also differs from *G.gemma* by having a larger and globosely ovate shell (height 13.3–16.0; Table 1), more turreted spire, convex whorls, vertically elongated aperture, and deeper suture. Whereas *G.gemma* possesses a smaller and slender shell (height 8.7–9.2; Table 1), spire high conical, flatly convex whorls, more rounded aperture, and shallow suture.

Additionally, this new species differs from *G.crassilabris* s. s. from India and its variety from Rakhine State, Myanmar, in having a smoother shell, obtuse, and lower and broader spire. While *G.crassilabris* s. s. (Fig. 5E, F) displays stronger striations and a narrowly pointed spire (Blanford 1865).

#### Remark.

So far, the genitalia of eight *Glessula* species has. The terminal part of the male genitalia of *G.mandalayensis* sp. nov. (Fig. 9A) clearly differs from these eight species in having no flagellum, while a flagellum is well-developed with a comb-like shape in the others (Table 3). However, we examined only a single specimen; therefore, the extent of variability within the species remains unknown.

### ﻿﻿15 Glessula orophila

Taxon classificationAnimaliaStylommatophoraSubulinidae

(Reeve, 1849)

EF996A66-19ED-5D0C-897E-533C85FBC357

Fig. 10A

Achatina orophila Reeve, 1849a: Achatina, pl. 19, species 105. Type locality: Neilgherry Hills, India [Nilgiri Hills, India]; Colombo, Ceylon. Benson 1860: 465.
Oleacina (Electra) orophila —Adams and Adams 1855: 106.
Cionella (Glessula) orophila —von Martens 1860: 254.
Stenogyra (Glessula) orophila —Nevill 1881: 137, pl. 5, fig. 19.
Glessula orophila —Beddome 1906: 168. Pilsbry 1909: 79, pl. 10, fig. 10. Gude 1914: 423. Raheem et al. 2014: 128, fig. 83a.

#### Type specimen.

The type specimen could not be located from the UMZC and the NHMUK collections (Raheem et al. 2014; Preece et al. 2022).

#### Other material.

**Figure 10.**
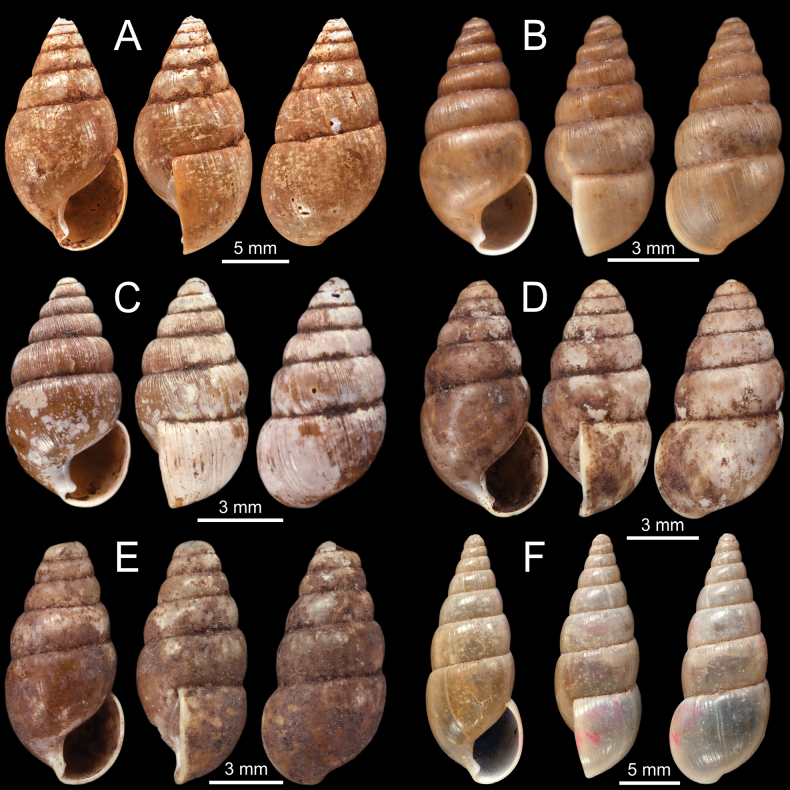
**A***Glessulaorophila*, specimen NHMUK ex. Blanford collection from Mahableshwar **B, C***Glessulapeguensis*, specimen NHMUK 1909.3.15.8 from Thyet-Myo **D, E***Glessulaperlevis*, syntypes NHMUK 1906.5.5.89 from Shan Hills, Burma **F***Glessulaponsiensis*, syntypes NHMUK 1989042 from Ponsee.

NHMUK (3 shells + 2 juveniles; Fig. 10A) ex. Blanford collection from Mahableshwar.

#### Diagnosis.

Shell ovate conical and regularly attenuated; spire low conical; apex rounded; subsequent whorls with very weak irregularly spaced radial ridges, and fine radial groves present. Suture shallow and whorls flattened. Last whorl large and ovate; aperture ovate; columella concave and truncated.

#### Distribution.

This species was first introduced from India and later reported from Sri Lanka and Pegu [Bago Region, Myanmar] (Gude 1914: 423).

#### Remarks.

*Glessulaorophila* is listed in the doubtful name by Raheem et al. (2014). No Myanmar material has been examined, and only the literature record is known.

### ﻿﻿16 Glessula peguensis

Taxon classificationAnimaliaStylommatophoraSubulinidae

(Blanford, 1865)

F0FF3178-B782-570F-8116-65B71C7A87E9

Fig. 10B, C

Achatina peguensis Blanford, 1865: 78. Type locality: Irawady valley, Pegu [Ayeyarwady valley, Bago Region, Myanmar]. Pfeiffer 1868: 228. Hanley and Theobald 1874: 41, pl. 102, fig. 6.
Glessula peguensis —Theobald and Stoliczka 1872: 334. Pfeiffer and Clessin 1881: 331. Beddome 1906: 171. Pilsbry 1909: 99, pl. 13, fig. 12. Gude 1914: 438. Godwin-Austen 1920: 55, 56, pl. 162, figs 20, 21.
Stenogyra (Glessula) peguensis —Nevill 1878: 171.

#### Type specimen.

The type specimen could not be located in the NHMUK collection.

#### Other material.

NHMUK 1909.3.15.8 (3 shells; Fig. 10B, C) ex. Blanford collection from Thyet-Myo. NHMUK 20230920 ex. H.B.F. collection (4 shells) from Thyet Myo. SMF 235657/2 (2 shells) from Pegu.

#### Diagnosis.

Shell oblong ovate and regularly attenuated; spire high conical; apex rounded and blunt; subsequent whorls with prominent regularly spaced radial ribs. Suture deeply impressed and whorls convex. Aperture widely ovate; columella strong, concave, and truncated.

#### Distribution.

*Glessulapeguensis* is distributed in Bago Region, Magway Region, and Rakhine State in Myanmar, as well as in Chittagong, Bangladesh (Gude 1914; Godwin-Austen 1920).

#### Remarks.

The specimen from ‘Thyet-myo’, which is present in the Blanford collection with the original label as ‘authentic’, is illustrated herein as an example of this species. According to Godwin-Austen (1920: 56), this species is commonly found in Pegu.

The original type locality of this species was from a broad geographical range, ‘Pegu’, and the museum label stated Thyet-Myo as the collection locality. The reference to ‘Pegu’ and ‘Thyet-Myo’ on the original label needs to be clarified as to whether they refer to the same area or if there is a specific location in ‘Thyet-Myo, Pegu’. The current administration area now includes ‘Thyet-Myo’ [Thayet Myo] as a district of the Magway Region located in central Myanmar, formerly part of ‘Pegu, Lower Burma’ during the British colonial period. Furthermore, Godwin-Austen (1920: 56) stated that additional specimens from ‘Arakan near Tongoop’ (No. 262.06.2.2) tended to have a larger shell size than the type specimen.

### ﻿﻿17 Glessula perlevis

Taxon classificationAnimaliaStylommatophoraSubulinidae

Godwin-Austen, 1920

0EA43DC3-2B36-5EAF-BD89-F8D6B19F895F

Fig. 10D, E

Glessula perlevis Godwin-Austen, 1920: 59. Type locality: Shan Hills [Shan State, Myanmar].

#### Type specimens.

***Syntypes***NHMUK 1906.5.5.89 [re-registered in error as 1986023] (5 shells; Fig. 10D, E) ex. Godwin-Austen collection from Shan Hills, Burma.

#### Diagnosis.

Shell oblong ovate and regularly attenuated; spire high conical; apex rounded and blunt; subsequent whorls nearly smooth with very weak growth lines, and somewhat strong irregularly spaced ridges near suture. Suture impressed and whorls slightly convex. Aperture ovate; columella concave and truncated.

#### Distribution.

This species is known only from the type locality in the Shan Hills near Mandalay. The type locality noted by Godwin-Austen seems ambiguous because it was described as ‘probably comes from the Shan State near Mandalay collected by Fedden’ (Godwin-Austen, 1920: 59).

#### Remarks.

Godwin-Austen (1920) described this species based on five specimens in the Blanford collection. The species description included one set of shell measurements, the number of specimens examined and the W.T. Blanford catalogue number ‘Type No. 89.06.5.5’ (NHMUK 1906.5.5.89), and there was no illustration of the shell. The NHMUK collections contain a type specimen lot consisting of five shells (2 matures + 3 immatures) with an original label stating ‘Types’. The mature shells correspond well with the shell dimensions given in the original description and are illustrated herein for the first time.

### ﻿﻿18 Glessula ponsiensis

Taxon classificationAnimaliaStylommatophoraSubulinidae

Godwin-Austen, 1920

76E4022A-8AEF-5B1D-BF5C-3AC31C442CA9

Figs 10F , 11A, B

Stenogyra (Glessula) pyramis var. major Nevill, 1878: 169 [nomen nudum]. Type locality: Ponsee.
Glessula ponsiensis —Godwin-Austen, 1920: 56, pl. 164, fig. 19. Type locality: Ponsee, Yunnan.

#### Type specimens.

**Figure 11.**
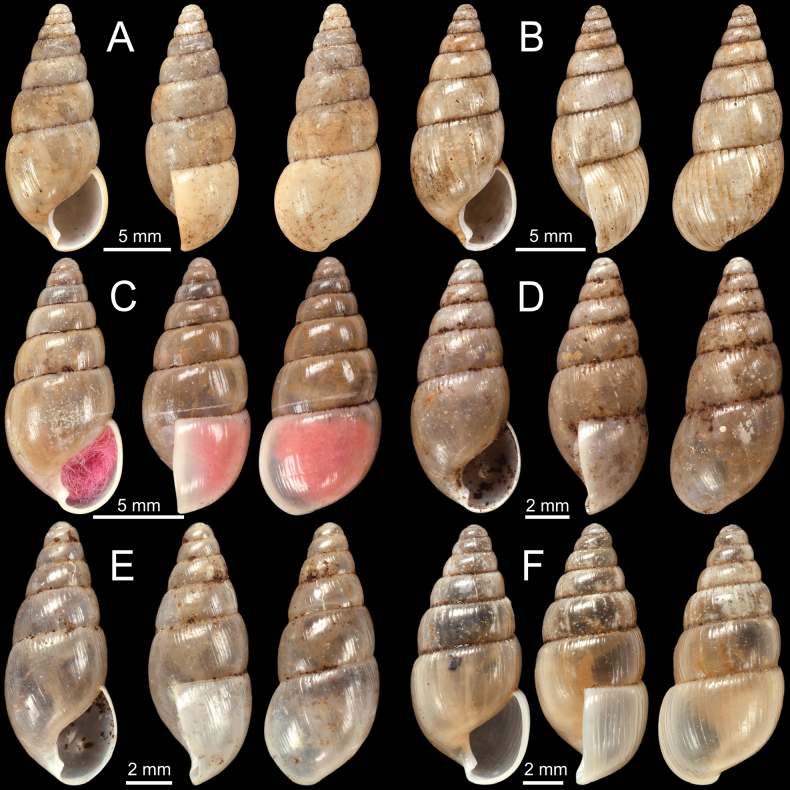
**A, B***Glessulaponsiensis***A** syntypes NHMUK 1989043 from Ponsee and **B** specimen NHMUK 1912.4.16.747 from Ponsee, Yunnan **C–E***Glessulawoodthorpei*, syntypes NHMUK 1903.7.1.1628 from Shan States **F***Glessulayuangensis*, holotype NHMUK 1903.7.1.1312 from Yuang Ha, Shan States.

***Syntypes***NHMUK 1989042 (1 shell; Fig. 10F) and NHMUK 1989043 (3 shells; Fig. 11A), both specimen lots collected by J. Anderson from Ponsee.

#### Other material.

NHMUK 1912.4.16.747 (2 shells; Fig. 11B) ex. Beddome collection from Ponsee, Yunnan.

#### Diagnosis.

Shell oblong turreted and regularly attenuated; spire high conical; apex rounded and blunt; subsequent whorls nearly smooth with fine growth lines, and strong, equally spaced radial ridges near suture. Suture impressed and whorls slightly convex. Aperture ovate; columella strong, concave, and truncated.

#### Distribution.

*Glessulaponsiensis* was first described from Ponsee, Yunnan, and later discovered in Bhamao, Kachin State, situated on the upper part of Ayeyarwady River.

#### Remarks.

The original description included a set of shell measurements and an illustration of the apex. Godwin-Austen (1920: 56) clearly states that this species was described based on specimens from the Indian Museum collection (now Zoological Survey of India (ZSI), Kolkata) collected by J. Anderson from Ponsee, which was misidentified as ‘Stenogyra (Glessula) pyramis Bs., var. major Nevill, 1878’. The name ‘ var. major’ by Nevill (1878: 169) is unavailable (ICZN 1999: Art. 12). Under the note of the species, Godwin-Austen states, ‘I am fortunate in getting the type-shells for examination’. It seemed that he received on loan the type specimen (or specimens that were examined by Nevill (1878: 169)). The NHMUK type collections contain a specimen lot with two registration numbers, M10905/2 and 10906/2, which are ex-Indian Museum collection numbers where the original label states ‘Stenogyra (Glessula) pyramis Benson, var. major’, collection of ‘Dr. J. Anderson’ and with collection locality from ‘Ponsee’. Interestingly, a species-group name was struck through and rewritten with red ink by Godwin-Austen, handwritten as ‘*ponsiensis*, G-A.’ and with ‘Type and three other one takes for B.M.’. It is probable that these specimens were presented to Godwin-Austen for examination. The specimen NHMUK 1989042 (1 shell has blue wool inside the shell; Fig. 10F) has a subsequent label stating ‘holotype’, and NHMUK 1989043 (3 shells; Fig. 11A) originates from the same Nevill type lot and has a subsequent label stating ‘paratypes’. This is not a valid holotype designation (ICZN 1999: Arts 73.1, 73.2 and Recommendation 73F) and these specimens are considered as the syntypes, illustrated herein for the first time.

### ﻿﻿19 Glessula woodthorpei

Taxon classificationAnimaliaStylommatophoraSubulinidae

Godwin-Austen, 1920

E2CE7E50-CA86-5439-85F0-56C195CB4AB9

Fig. 11C–E

Glessula woodthorpei Godwin-Austen, 1920: 58, pl. 162, fig. 19. Type locality: Shan States [Shan State, Myanmar].

#### Type specimens.

***Syntypes***NHMUK 1903.7.1.1628 [re-registered in error as 1986051] (9 shells; Fig. 11C–E) collected by Woodthorpe ex. Godwin-Austen collection from Shan States.

#### Other material.

NHMUK 1903.7.1.3655 (2 shells) collected by Woodthorpe ex. Godwin-Austen collection from Siam N.W. Boundary.

#### Diagnosis.

Shell oblong conical and regularly attenuated; spire high conical; embryonic whorls rounded; subsequent whorls nearly smooth and equally spaced radial ridges appeared near suture. Suture impressed and whorls slightly convex. Aperture ovate; columella strong, concave, and truncated.

#### Distribution.

This species is known from Shan State, Myanmar with an additional record from Thailand.

#### Remarks.

Godwin-Austen (1920) indicated that nine specimens with the catalogue number ‘Type No. 1682 B.M.’ were examined. The species description included one set of measurements and an illustration of one shell. The NHMUK type collections contain a lot of nine specimens from the Woodthorpe collection with a label in Godwin-Austen’s handwriting stating ‘Type’. The measurements of the specimen with red wool inside the shell are close to the measurements given in the original description. It likely corresponds to the illustration of the species provided in the original description and it herein figured (Fig. 11C) along with other shells (Fig. 11D, E) from the same syntype lot.

Godwin-Austen separately mentioned (1920: 58) another bleached specimen lot NHMUK 1903.7.1.3655 (2 shells) from Thailand. However, this specimen lot does not form part of the type series of this species.

### ﻿﻿20 Glessula yuangensis

Taxon classificationAnimaliaStylommatophoraSubulinidae

Godwin-Austen, 1920

9A8FE930-2EA7-5D4C-BC51-C955183488E8

Fig. 11F

Glessula yuangensis Godwin-Austen, 1920: 59, pl. 162, fig. 18. Type locality: Yuang Ha, Siam Boundary [Mong Yawng Township, Tachileik District, Shan State, Myanmar].

#### Type specimen.

***Holotype***NHMUK 1903.7.1.1312 [re-registered in error as 1986052] (Fig. 11F) collected by Woodthorpe ex. Godwin-Austen collection from Yuang Ha, Shan States.

#### Diagnosis.

Shell oblong turreted and regularly attenuated; spire high conical; apex rounded and blunt; subsequent whorls with prominent wide spaced radial ribs, and radial groves present. Suture impressed and whorls slightly convex. Aperture ovate; columella strong, concave, and truncated.

#### Distribution.

The species is currently known only from its type locality.

#### Remarks.

Godwin-Austen clearly stated that this species was described based on only one specimen collected by R. Woodthorpe. The description included a set of shell dimensions and a shell illustration. There is one Godwin-Austen specimen lot, NHMUK 1903.7.1.1312, that has an original label stating ‘Type’ and is from the Woodthorpe collection. We recognise this specimen as the holotype fixed by monotypy. Additionally, Godwin-Austen seemed to consider another specimen lot, ‘No. 1156 B.M.’ (NHMUK 1903.7.1.1156) from Kentung State [Kengtung Township, Shan State], as a distinct entity and it is therefore excluded from the type series.

Originally, the type locality was said to be from ‘Yuang Ha, Siam Boundary’; however, this town currently is found in the Shan State, Myanmar. The original type locality is now Mong Yawng Township, Shan State (~ 21°10'42.7"N, 100°21'24.3"E), Myanmar, with a further record from Kengtung, Shan State (Godwin-Austen 1920: 59).

### ﻿﻿Genus Opeas

Taxon classificationAnimaliaStylommatophoraSubulinidae

Albers, 1850

51D77543-C0BF-5001-AA2F-9B616E685C5E

Bulimus (Opeas) Albers, 1850: 175, 176.
Stenogyra (Opeas) —von Martens 1860: 265, 266.
Opeas —Fischer and Crosse 1877: 592. Pilsbry 1906: 122. Gude 1914: 354. Zilch 1959: 351, 352. van Benthem Jutting 1952: 378. Schileyko 1999: 492.

#### Type species.

*Helixgoodallii* Miller, 1822 [junior homonym of Férussac (1821)] accepted as *Opeashannense* (Rang, 1831), subsequent designation by von Martens in Albers (1860: 265).

#### Diagnosis.

Shell small, slender, and conical; spire high, turreted, cylindrically, and gradually attenuated; embryonic whorls smooth; subsequent whorls with fine radial striations or growth lines. Aperture vertical, narrow, oblong, columellar margin expanded, and columella straight or concave. Umbilicus narrowly opened or closed. Penis cylindrical tube with short epiphallus, and flagellum and penial sheath absent; vagina long ~ 1/2 of penis length.

#### Remarks.

*Opeas* and *Tortaxis* Pilsbry, 1906 generally resemble one another in having slender conical or cylindrical shells, nearly smooth to fine striations, and flatly convex whorls (Table 2). However, *Opeas* has a smaller size, straight columella, and mostly oblong aperture, whereas *Tortaxis* displays mostly larger size, nearly straight or slightly concave columella with a spiral fold, and more or less obliqued aperture. Likewise, *Opeas* is distinctly differentiated from *Bacillum* in that it is smaller in size, has a straight columella, fine shell sculptures and embryonic whorls which are narrowly rounded. In contrast, *Bacillum* exhibits a larger shell size, truncated and concave columella, stronger shell sculptures, and the embryonic whorls cylindrically rounded.

This genus is distributed in tropical and subtropical regions in Europe, Asia, Africa, and North America, and comprises nearly 200 species (Schileyko 1999; MolluscaBase 2023). In Myanmar, two species are recorded (Gude 1914).

### ﻿﻿21 Opeas filiforme

Taxon classificationAnimaliaStylommatophoraSubulinidae

von Möllendorff, 1894

EEE0A8A3-7A0E-5512-98D6-8F64E0B8169E

**Figure 12.**
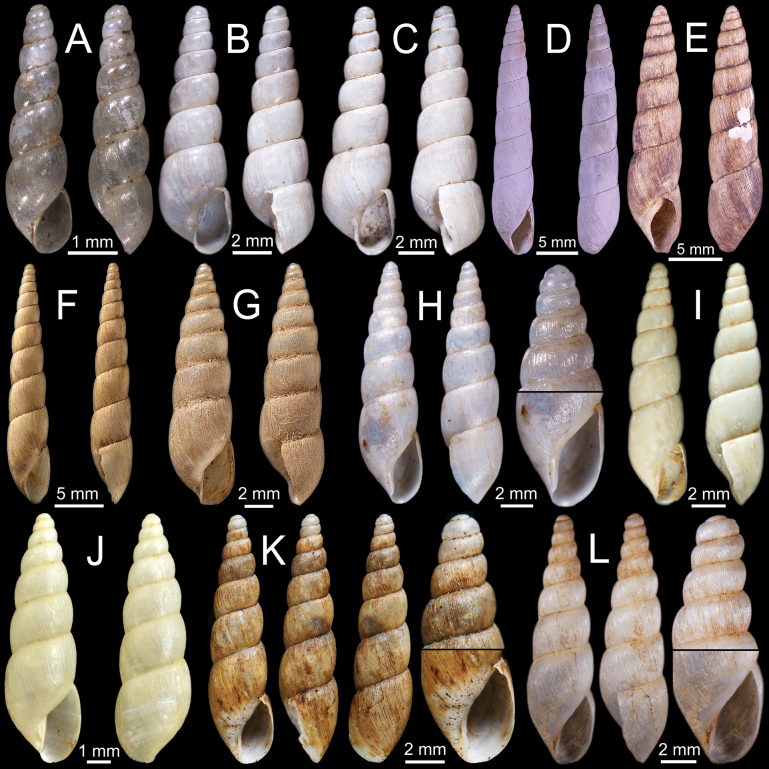
**A–C***Opeasfiliforme***A** lectotype SMF 227532 from Gulf von Siam: Samui-Inseln **B, C** specimen CUMZ 13074 from Tanintharyi Region, Myanmar **D***Paropeasswettenhami*, syntype MNHN-IM-2000-38930 from Malacca **E***Paropeastchehelense*, syntype MNHN-IM-2000-38932 from Pérak, Mont Tchéhèl **F***Paropeasterebralis*, syntype NHMUK 1888.12.4.1081 from Shan States, Burma **G, H***Paropeasturricula***G** syntypes NHMUK 1859.8.1.10 from Siam and **H** specimen CUMZ 13076 from Tanintharyi Region, Myanmar **I–L***Paropeaswalkeri***I** syntypes UMZC I.103115.A from Andaman Island (after Preece et al. 2022: fig. 56e) **J** specimen NHMUK 1885.2.18.13–18 from Viper Id. Andamans **K** specimen CUMZ 13077 from Taunggyi Township, Shan State, Myanmar with embryonic whorls and aperture and **L** specimen CUMZ 13078 from Kalaw Township, Shan State, Myanmar with embryonic whorls and aperture. Photographs: V Gojšina & B Páll-Gergely (**A**), P Bourguignon (**D, E**).

Fig. 12A–C , Table 1

Opeas filiforme von Möllendorff, 1894: 151, pl. 16, fig. 11. Type locality: Golf von Siam: Samui-Inseln [Samui Island, Suratthani Province, Thailand]. Pilsbry 1906: 161, pl. 19, fig. 22. Zilch 1973: 120.
Prosopeas filiforme —Panha 1998: 32.

#### Type specimens.

***Lectotype*** SMF 227532 (Fig. 12A) von Möllendorff ex. C. Roebelen from Gulf von Siam: Samui-Inseln, designated in Zilch (1973: 120). ***Paralectotype*s** SMF 145657/7 (7 shells), SMF 227533/7 (7 shells).

#### Other material.

Phra (Buddha) Cave, Tanintharyi Region, Myanmar (11°14'01.5"N, 99°10'42.8"E): CUMZ 13074 (15 shells; Fig. 12B, C).

#### Description.

Shell slender and conical, translucent, whitish colour, and with 7½–8 whorls. Apex rounded; protoconch ~ 2 whorls, rounded and smooth surface; subsequent whorls generally nearly smooth with fine growth lines. Spire gradually and cylindrically tapering and largely turreted; whorls flatly convex and last whorl largest; suture deep and weakly crenulated. Aperture oblong; peristome thin; columella straight; columellar margin slightly expanded. Umbilicus narrowly opened to closed.

#### Distribution.

This species occurs in peninsular Thailand and is newly recorded from the Tanintharyi Region, southern Myanmar.

#### Remarks.

New discovered of this species were found in Tanintharyi, which borders the type locality of Thailand. The type specimen (Fig. 12A) possesses less turreted and more convex whorls than our specimens; however, this differentiation is herein considered an intraspecific variation. Compared to *O.innocens* Preston, 1910 from Mawlamyine, *O.filiforme* from Tanintharyi can generally be differentiated by its slender and more attenuated shell, slightly convex whorls, and turreted spire. However, a comprehensive examination of *O.innocens* specimens from the type locality is necessary to confirm this distinction.

### ﻿﻿22 Opeas innocens

Taxon classificationAnimaliaStylommatophoraSubulinidae

Preston, 1910

E2D34660-C9E4-5A2C-BEFF-050B3E8F1263

Opeas innocens Preston, 1910: 33, 34, fig. 2. Type locality: Khayon Cave, near Moulmein, Lower Burma [Khayon or Farm Cave, Mawlamyine Township, Mon State, Myanmar]. Gude 1914: 358.

#### Distribution.

This species is known only from the type locality.

#### Remarks.

No new specimens were found during our sampling conducted in Mawlamyine, Mon State. Furthermore, the authenticated and type specimens could not be located in the NHMUK collection, and currently, this species is known solely from the original description.

### ﻿﻿Genus Paropeas

Taxon classificationAnimaliaStylommatophoraSubulinidae

Pilsbry, 1906

B7FF3714-175D-5C18-96E3-803512133513

Prosopeas (Paropeas) Pilsbry, 1906: 14. Zilch 1959: 353.
Paropeas —Naggs 1994: 175–191. Schileyko 1999: 508.

#### Type species.

*Bulimusacutissimus* Mousson, 1857 by original designation.

#### Diagnosis.

Shell slender and conical; spire high, turreted, and gradually attenuated; embryonic and subsequent whorls with irregularly dense, fine, or coarse radial striations. Aperture oblique, narrow or broadly ovate; columella concave; columellar margin expanded or not expanded. Penial simple, relatively long, slender, epiphallus short, flagellum absent, and penial sheath present; vagina ~ 1/2 of penis length and wider than penis.

#### Remarks.

*Paropeas* can be distinguished from *Bacillum* and *Rishetia* by its irregularly coarse radial striations throughout the shell, less concave or straighter columella, and narrowly tapering and pointed embryonic whorls (Table 2). While *Bacillum* has stronger and more evenly spaced radial striations, deeper concave and truncate columella, and cylindrically tapering and obtuse embryonic whorls. Likewise, *Rishetia* has a glossy and smoother shell, broader whorls, more concave columella, and rounded and wider aperture (Pilsbry 1906; Gude 1914; Naggs 1994). In terms of genitalia, *Rishetia* is obviously distinct from *Paropeas* by having a tubular-shaped flagellum, while *Paropeas* has no flagellum (Naggs 1994; Schileyko 1999).

*Paropeas* and *Prosopeas* are very similar in terms of shell form and sculpture, but *Paropeas* possess stronger and more compact striations on embryonic whorls (Gude 1914; Naggs 1994). However, distinguishing between these two genera is still challenging, and precise identification requires further evidence, such as data on genitalia and molecular phylogeny.

This genus is mainly distributed in Southeast Asia. Seven recognised species are present, four are reported in Myanmar (Gude 1914; Naggs 1994; Schileyko 1999; MolluscaBase 2023).

### ﻿﻿23 Paropeas swettenhami

Taxon classificationAnimaliaStylommatophoraSubulinidae

(de Morgan, 1885)

01940F4C-74CD-58B5-8013-0427ECF65D0B

Fig. 12D

Stenogyra swettenhami de Morgan, 1885: 389, pl. 6, fig. 6. Type locality: G. Tchöra, près ďIpoh (Kinta) [Gunung Cheroh, Ipoh, Perak State, Malaysia].
Prosopeas swettenhami —Pilsbry 1906: 32, pl. 4, figs 11, 12. Laidlaw 1933: 216.
Paropeas swettenhami —Naggs 1994: 188.

#### Type specimen.

***Syntype***MNHN-IM-2000-38930 (1 shell; Fig. 12D) from Malacca, G. Tchéhèl.

#### Diagnosis.

Shell slender, elongate turreted and rapidly attenuated; apex pointed; subsequent whorls coarse with dense and fine radial striae. Suture wide and shallow, and whorls flattened. Aperture elongate ovate; columella concave.

#### Distribution.

This species has been reported from Malaysia and Shan State, Myanmar (Naggs 1994).

#### Remarks.

de Morgan (1885) describes *P.swettenhami* and *P.tchehelense* consecutively in the same publication and from a very close geographical area within Perak State, Peninsular Malaysia. Then von Möllendorff (1891: 337) suggests ‘*Stenogyraswettenhami* de Morgan, 1885’ as a slight variation of *P.tchehelense*; therefore, some subsequent authors recognise the former as a synonym of the latter (i.e., Maassen 2001; MolluscaBase 2023). In contrast, Pilsbry (1906: 32) argues that they differ in shell shape and structure. We have examined the syntypes of both species and agree with Pilsbry (1906) that *P.swettenhami* (Fig. 12D) differs from *P.tchehelense* by having a slimmer shell, narrower aperture, cylindrical and rounded embryonic whorls. *Prosopeastchehelense* (Fig. 12E) possesses a large, broad shell, wide aperture, convex, and rounded embryonic whorls.

No new materials of this species were found in this survey, and the type specimen is illustrated herein. Naggs (1994) provisionally reported the occurrence of *P.swettenhami* from Shan State, Myanmar. Considering the vast geographical distance between Perak and Shan State, it may be that the specimens Naggs (1994) regarded as *P.swettenhami* are, in fact, *P.terebralis* or another species. However, collecting topotypical material from Shan State, Myanmar, is necessary to confirm the existence of this species in Myanmar and the systematic relationship with *P.tchehelense*.

### ﻿﻿24 Paropeas terebralis

Taxon classificationAnimaliaStylommatophoraSubulinidae

(Theobald, 1870)

81E1FEF2-1A07-57D0-996C-ED92B8B7204D

Fig. 12F

Stenogyra (Opeas) terebralis Theobald, 1870: 401. Type locality: Shan States [Shan State, Myanmar]. Nevill 1878: 166. Pfeiffer and Clessin 1881: 321.
Bulimus (Stenogyra) terebralis —Pfeiffer 1876: 133.
Prosopeas terebrale —Pilsbry 1906: 31. Gude 1914: 363.
Paraopeas terebrali s—Naggs 1994: 188.

#### Type specimen.

***Syntype***NHMUK 1888.12.4.1081 (1 shell; Fig. 12F) ex. Theobald collection from Shan States, Burma.

#### Diagnosis.

Shell slender, elongate turreted and rapidly attenuated; apex obtuse; subsequent whorls coarse with dense growth lines throughout. Suture wide and shallow, and whorls slightly convex. Aperture elongate ovate and narrow; columella curved; peristome little acute.

#### Distribution.

This species is only known from the type locality.

#### Remarks.

No recent material of this species was collected in this survey, and the syntype is illustrated here for the first time. Gude (1914) recognised this species as belonging to the *Prosopeas*, but Naggs (1994) provisionally transferred this species to the *Paropeas* after comparing it with the type specimen.

*Paropeasterebralis* can be distinguished from *P.swettenhami* by its broader and more rounded embryonic whorls, deeper suture and slightly convex whorls, and coarse shell surface with dense growth lines. Whereas *P.swettenhami* possesses narrower and pointed embryonic whorls, shallower suture and flattened whorls, and shell surface with dense and fine radial striae.

### ﻿﻿25 Paropeas turricula

Taxon classificationAnimaliaStylommatophoraSubulinidae

(von Martens, 1860)

F476D5A6-96EC-5378-B44B-3460859EFC4C

Fig. 12G, H , Table 1

Stenogyra turricula von Martens, 1860: 9. Type locality: Siam [Thailand]. von Martens 1867: 82, 83, pl. 22, fig. 7.
Paropeas turricula —Naggs 1994: 188. Maassen 2001: 81.
Prosopeas turricul a—Pilsbry 1906: 30, 31, pl. 3, figs 95, 96. Inkhavilay et al. 2019: 52.

#### Type specimens.

***Syntypes***NHMUK 1859.8.1.10 (3 shells; Fig. 12G) from Siam.

#### Other material.

Phra (Buddha) Cave, Tanintharyi Region, Myanmar (11°14'01.5"N, 99°10'42.8"E): CUMZ 13075 (1 shell). The limestone karsts located close to Lampane Village, Tanintharyi Region, Myanmar (11°40'18.1"N, 99°13'30.1"E): CUMZ 13076 (2 shells; Fig. 12H).

#### Description.

Shell slender, conical, translucent, whitish colour, and with 8–8½ whorls. Apex rounded; protoconch ~ 2 whorls, turreted and with nearly smooth to fine radial striations; subsequent whorls with dense and fine radial striations. Spire high, turreted, gradually, and cylindrically tapering; whorls flatly convex, last three whorls nearly equal; suture deep. Aperture narrowly ovate and elongate; peristome thin; columella truncated and concave; columellar margin slightly expanded. Umbilicus narrowly opened to closed.

#### Distribution.

This species was first described in Thailand, then subsequently recorded in Laos and Malaysia (Naggs 1994; Maassen 2001; Inkhavilay et al. 2019), and is here reported from the Tanintharyi Region, Myanmar.

#### Remarks.

*Paropeasturricula* can be differentiated from *P.tchehelense* by its slender shell, cylindrically turreted spire, and finer striations, while *P.tchehelense* has a broader and larger shell, a more rounded and broader spire, and stronger radial striations. This species can also be separated from *P.terebralis* from Shan State by having finer sculpture, flatly convex whorls, shallower suture, and embryonic whorls that are more turreted with nearly smooth to weaker striations, whereas *P.terebralis* displays a more elongated shell, thicker sculptures, more convex whorls, deeper suture, and embryonic whorls more rounded with stronger striations. *Paropeasswettenhami* has a larger shell, stronger striations, and wider and more deeply concave columella.

### ﻿﻿26 Paropeas walkeri

Taxon classificationAnimaliaStylommatophoraSubulinidae

(Benson, 1863)

5A6DC5FB-57D5-51CC-951C-EB888DF23E29

Fig. 12I–L , Table 1

Spiraxis walkeri Benson, 1863: 90. Type locality: ad Portum Blair [Port Blair, Andaman Islands, India]. Pfeiffer 1868: 189. Hanley and Theobald 1873: 34, pl. 79, fig. 4.
Opeas walkeri —Theobald 1870: 395. Godwin-Austen 1895: 443. Blanford 1903: 280.
Stenogyra (Opeas) walker i—Nevill 1878: 165.
Stenogyra [Spiraxis (Euspiraxis)] walkeri—Pfeiffer and Clessin 1881: 323.
Prosopeas walkeri —Pilsbry 1906: 29, 30, pl. 6, fig. 70. Gude 1914: 363, 364. Ramakrishna et al. 2010: 183. Preece et al. 2022: 130, 131, fig. 56e.

#### Type specimens.

***Syntypes***UMZC I.103115.A (5 shells; Fig. 12I, after Preece et al. 2022: fig. 56e) ex. R. McAndrew collection from Andaman Island.

#### Other material.

NHMUK 1885.2.18.13–18 ex E.S. Berkeley collection Viper Id. Andamans: (6 shells; Fig. 12J). Aik Kham Cave, Taunggyi Township, Taunggyi District, Shan State, Myanmar (20°49'07.0"N, 97°13'42.0"E): CUMZ 13077 (3 shells; Fig. 12K). Ywangan Village, near Lin Way Monastery, Kalaw Township, Taunggyi District, Shan State, Myanmar (21°13'43.3"N, 96°33'19.2"E): CUMZ 13078 (2 shells; Fig. 12L).

#### Description.

Shell slender, conical, translucent, whitish to pale yellowish colour, and with 8–8½ whorls. Apex rounded; protoconch ~ 2 whorls, rounded and with fine radial striations on entire whorls. Spire high, gradually tapering, and turreted; whorls flatly convex and last three whorls nearly equal; suture narrow and deep. Aperture narrowly ovate and elongate; peristome thin; columella straight or slightly concave; columellar margin simple to slightly expanded. Umbilicus narrowly opened to closed.

#### Distribution.

This species was originally described from the Andaman Islands, India, and was later recorded in Shan State, Myanmar and Thailand (Gude 1914; Panha 1998).

#### Remarks.

In Myanmar, *Paropeasterebralis* and *P.walkeri* are both recorded from Shan State. However, *P.walkeri* possesses broader, fewer, and more convex whorls, a straight columella with a slightly reflected columellar margin, weaker radial striations, shallow suture, and embryonic whorls rounded. By contrast, *P.terebralis* has slimmer, higher, and flatter whorls, more concave columella, strong radial striations, deeper suture, and more pointed embryonic whorls. Additionally, *P.walkeri* also differs from *P.turricula* by its more convex whorls, straight columella, slightly reflected columellar margin, finer and crowded radial striations, deeper suture, and embryonic whorls rounded with stronger radial striations. Furthermore, *P.walkeri* can be differentiated from *P.swettenhami* by its slenderer shell, convex and closely grow whorls, deeper suture, more rounded protoconch whorls, distinctly turreted spire, and straight columella. *Prosopeasswettenhami* displays a broader shell, flatter whorls, shallow suture, convex protoconch whorls, less turreted spire, and more concave columella.

Among the specimens examined from Shan State, we observed shell variations, such as the columellar margin being either expanded or not expanded and the columella being straight or slightly concave. In addition, the specimen identified as *P.walkeri* from the Andaman Islands (Fig. 12J) exhibits a broader shell and a more expanded columellar margin compared to the syntype specimen (Fig. 12I).

### ﻿﻿Genus Rishetia

Taxon classificationAnimaliaStylommatophoraSubulinidae

Godwin-Austen, 1920

55233A14-92DE-5378-A4A9-6E54388C321F

Glessula (Rishetia) Godwin-Austen, 1920: 7.
Rishetia —Schileyko 1999: 532. Raheem et al. 2014: 138, 139. Budha et al. 2017: 137. Preece et al. 2022: 127.

#### Type species.

*Achatinatenuispira* Benson, 1836 by original designation.

#### Diagnosis.

Shell slender and conical; spire high, turreted, and regularly attenuated; embryonic whorls smooth or with striations and subsequent whorls have thick or fine and equally or irregularly spaced radial ribs. Aperture oblique, narrow to broad, and ovate shape; columella concave and truncated; columellar margin not expanded. Penis large, thick, and moderately long; epiphallus relatively long; flagellum present with tubular shape; epiphallic caecum present; vagina enlarged, short ~ 1/2 of penis length.

#### Remarks.

Originally, Godwin-Austen (1920) proposed *Rishetia* as a subgenus of *Glessula*, which Schileyko (1999) raised to the generic level, followed by Raheem et al. (2014), Budha et al. (2017), and Preece et al. (2022). *Rishetia* can be distinguished from *Glessula* by having a slender elongate-conical shell with a tubular-shaped flagellum and epiphallic caecum present, while *Glessula* processes an ovate-conical shell with a comb-like flagellum and epiphallic caecum absent (Tables 2, 3).

*Rishetia* can generally be differentiated from *Bacillum* by having a narrowly attenuated shell, convex and smaller apex, straight columella, and weak striations; *Bacillum* has a cylindrical shell, large and rounded apex, more concave columella, and stronger radial striations (Table 2). More information on the genitalia as well as a molecular phylogeny based on multiple species will clarify the systematic relationship of these two genera.

This genus has been documented in India, Sri Lanka, Bangladesh, and Nepal, with its presence in Southeast Asia limited to Myanmar (Godwin-Austen 1920; Schileyko 1999; Raheem et al. 2014; Budha et al. 2015, 2017). Currently, 23 species are recognised; among these, eleven species and subspecies have been documented from Myanmar (Godwin-Austen 1920; MolluscaBase 2023).

### ﻿﻿27 Rishetia akouktoungensis

Taxon classificationAnimaliaStylommatophoraSubulinidae

(Godwin-Austen, 1920) comb. nov.

F3A7005B-B9D0-58F4-9051-E674F1F700D3

Fig. 13A–C

Glessula akouktoungensis Godwin-Austen, 1920: 55. Type locality: Akouktoung on Irawady, Pegu [Akauk Taung (hill), Pyay District, Bago Region, Myanmar].

#### Type specimens.

**Figure 13.**
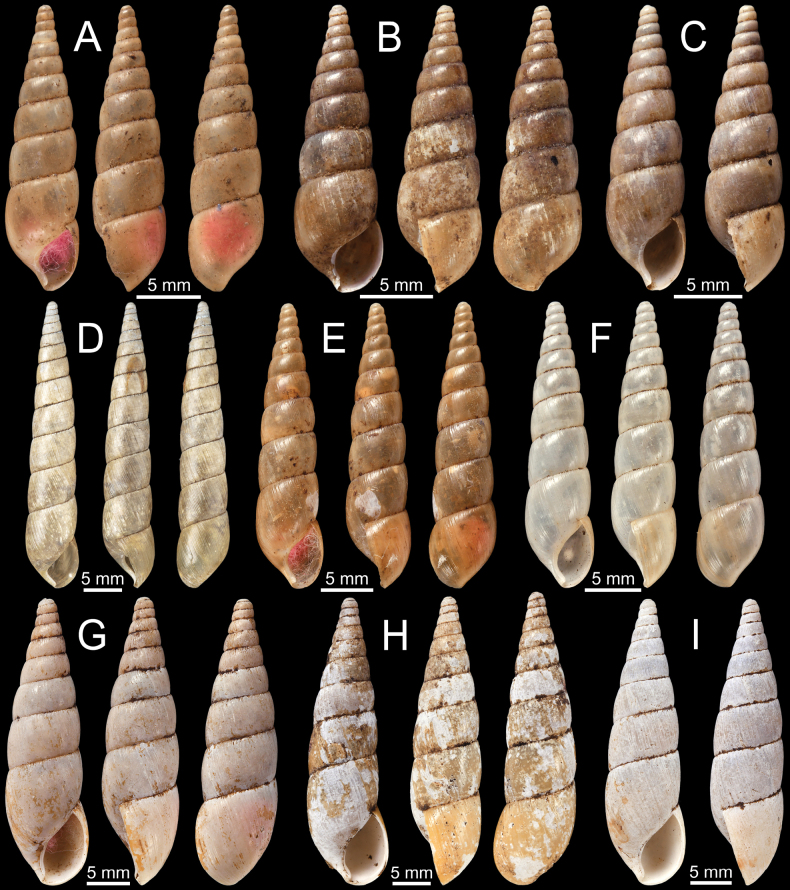
**A–C***Rishetiaakouktoungensis*, syntypes NHMUK 1906.1.1.2207 from Akouktoung, Pegu **D***Rishetiabaculina*, syntypes NHMUK 1909.3.15.9 from Kursiong, Darjiling **E, F***Rishetiabasseinensis*, syntypes NHMUK 1909.3.15.19 from Bassein **G–I***Rishetiaburrailensismaxwelli***G** holotype NHMUK 1903.7.1.1717/1 from Naga Hills, East of Kohima **H** specimen NHMUK 1988147 from Somra Tracts, Somra Khulen Post, west of Kyengdwen River, Burma and **I** paratypes NHMUK 1903.7.1.1717/2–4 from Naga Hills, East of Kohima.

***Syntypes***NHMUK 1906.1.1.2207 [re-registered in error as 1985138] (6 shells; Fig. 13A–C). ex. W.T. Blanford ex. Godwin-Austen collection from Akouktoung, Pegu.

#### Diagnosis.

Shell elongate, turreted, and regularly attenuated; apex rounded; subsequent whorls nearly smooth and with coarse radial ridges near suture. Suture impressed and whorls flattened convex. Aperture broadly ovate; columella strong, concave, and truncated.

#### Distribution.

The species is currently known from its type locality, in which the modern name is Akauk Taung [hill, ~ 18°30'33.7"N, 95°06'48.5"E], south of Htone Bo (or Tonbo) Village, Padaung Township, Pyay District, Bago Region].

#### Remarks.

The original description did not include an illustration, and only one set of measurements was given. Godwin-Austen’s description was based on one lot of specimens which clearly stated ‘Type No. 2207.06.1.1 B.M.’ (NHMUK 1906.1.1.2207). There are six specimens from the NHMUK type collection with the original label stating ‘Type’. The specimen that has a similar shell measurements as given in the original description and with pink wool inside the shell is figured herein (Fig. 13A). Other shells from the same syntype lot are also illustrated (Fig. 13B, C).

### ﻿﻿28 Rishetia baculina

Taxon classificationAnimaliaStylommatophoraSubulinidae

(Blanford, 1871) comb. nov.

D6793898-3302-511C-B154-F5C93C3A4B88

Fig. 13D

Glessula baculina Blanford, 1871: 43, 44, pl. 2, fig. 6. Type locality: Khersiong Himalayae Sikkimensis. Beddome 1906: 160. Gude 1914: 379, 380. Godwin-Austen 1920: 14–16, pl. 159, fig. 7. Ramakrishna et al. 2010: 153.
Achatina (Electra) baculina —Hanley and Theobald 1873: 33, pl. 78, fig. 6.
Achatina baculina —Pfeiffer 1876: 291.
Stenogyra (Glessula) baculin a—Nevill 1878: 170.
Stenogyra (Subulina) baculina —Pfeiffer and Clessin 1881: 327.
Glessula tenuispira var. baculina —Pilsbry 1909: 88, 89, pl. 9, fig. 2.

#### Type specimens.

***Syntypes***NHMUK 1909.3.15.9 [re-registered in error as 19850144] (3 shells; Fig. 13D) ex. Godwin-Austen ex. Blanford collection from Kursiong, Darjiling.

#### Diagnosis.

Shell slender, elongate turreted and rapidly attenuated; apex rounded; subsequent whorls with fine and equally spaced radial ribs throughout. Suture impressed and whorls slightly convex. Aperture obliquely ovate; columella strong, concave, and truncated.

#### Distribution.

This species was originally discovered in India and later reported from the Magway Region, Myanmar (Godwin-Austen 1920).

#### Remarks.

Godwin-Austen (1920: 15) stated that specimens identified to this species in the Beddome collection were collected from ‘Thyet Myo’ [Thayet District, Magway Region]. However, the Myanmar specimens show noticeable differences from the type specimens in having a more attenuated apex, and larger and broader aperture.

### ﻿﻿29 Rishetia basseinensis

Taxon classificationAnimaliaStylommatophoraSubulinidae

(Godwin-Austen, 1920) comb. nov.

0A00E2F8-6230-5E64-B88C-05A18645C894

Fig. 13E, F

Achatina pertenuis var. major Blanford, 1865: 79 [in part].
Glessula basseinensis —Godwin-Austen 1920: 54, pl. 161. fig. 3, pl. 164, fig. 12. Type locality: Bassein, Pegu and Pyema Khyoung, Bassein.

#### Type specimens.

***Syntypes***NHMUK 1909.3.15.19 [re-registered in error as 1985139] (3 shells; Fig. 13E, F) ex. Blanford ex. Godwin-Austen collection from Bassein, Pegu.

#### Diagnosis.

Shell slender, elongate turreted and regularly attenuated; apex rounded and blunt; subsequent whorls with fine and equally spaced radial ribs, which more prominent near suture. Suture impressed and whorls flattened convex. Aperture ovate; columella strong, concave, and truncated.

#### Distribution.

*Rishetiabasseinensis* appears to be restricted within Myanmar and is known only from the type locality. Based on the current administrative boundary, ‘Bassien (or Pathein), Pegus’ is in the Pathein Township, Pathein District, Ayeyarwady Region, rather than Pegu [Bago Region].

#### Remarks.

Godwin-Austen (1920: 53) stated that when examining the type series of ‘Glessulapertenuisvar.major’ ex. Blanford collection, he found it very different from the nominotypical taxon, and described this specimen lot under a different name, ‘*Glessulabasseinensis*’. In the original description, Godwin-Austen (1920: 54) provided two collection localities, namely ‘Bassein, Pegu’ and ‘Pyema Khyoung, Bassein’ and comparing three and six specimens, respectively. The description clearly states a catalogue number ‘Type No. 19.9.3.15 B.M.’ and includes an illustration of specimens from ‘Bassein, Pegu’. The NHMUK collection contains this specimen lot NHMUK 1909.3.15.19 consisting of three shells with an original label in Godwin-Austen’s handwriting stating ‘Type’ from the H.F. Blanford collection. The specimen with a similar shell measurement given in the original description and with pink wool inside the shell is figured herein (Fig. 13E). Additionally, one further shell from the same syntype lot is also illustrated (Fig. 13F).

The specimen lot NHMUK 1909.1.1.2208 is excluded from the type series of this species (see also under the *Rishetiapertenuismajor*).

### ﻿﻿30 Rishetia burrailensis maxwelli

Taxon classificationAnimaliaStylommatophoraSubulinidae

(Godwin-Austen, 1920) comb. nov.

04837403-AA6A-538D-92CF-1AD16EE01E7F

Figs 13G–I , 14A–C

Glessula (Rishetia) burrailensis , var. maxwelli Godwin-Austen, 1920: 45, 46, pl. 160, figs 5, 6. Type locality: Naga Hills, east of Kohima and Somra, Khulen Post. West of Kyendwin or Chindwin River, Upper Burma.
Glessula burrailensis , var. maxwelli—Godwin-Austen 1920: 61. Type locality: Somra Tracts, Somra Khulen Post, Upper Burma.

#### Type specimens.

***Holotype***NHMUK 1903.7.1.1717/1 (Fig. 13G) ex. Godwin-Austen collection from Naga Hills, East of Kohima. ***Paratypes***NHMUK 1903.7.1.1717/2–4 (3 shells; Fig. 13I) ex. Godwin-Austen collection from the location same as the holotype.

#### Other material.

**Figure 14.**
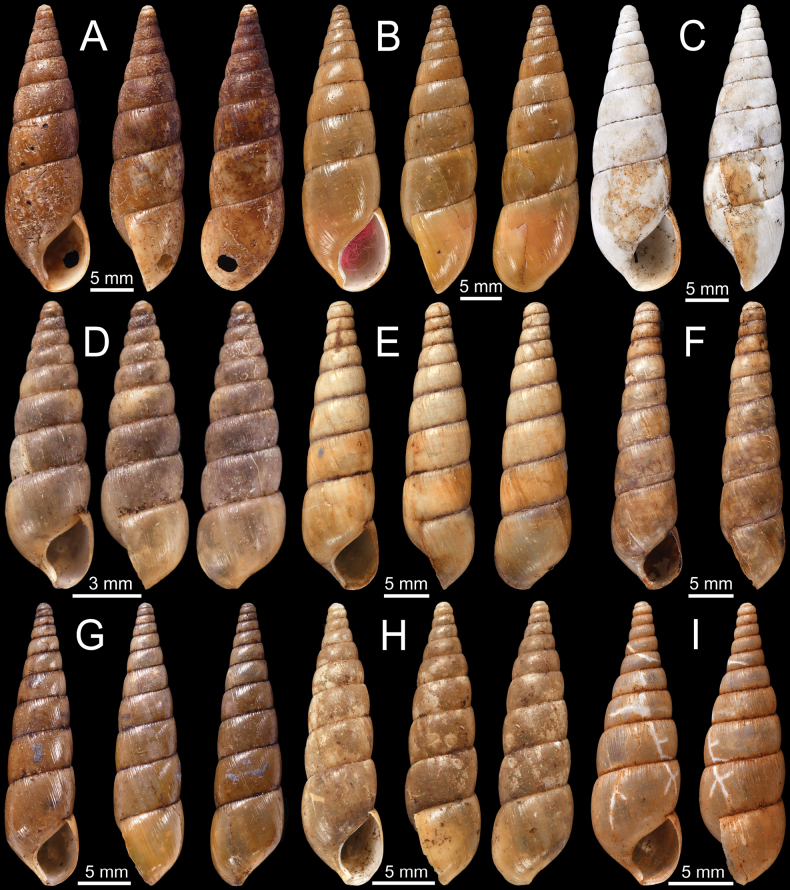
**A–C***Rishetiaburrailensismaxwelli***A, B** specimen NHMUK 1903.7.1.3742 from Somra Tracts, Somra Khulen Post, Burma and **C** specimen NHMUK 1903.7.1.3744 from Somra Tracts, Somra Khulen Post, Burma **D***Rishetiahastula*, neotype NHMUK 1906.1.1.880 from Darjiling **E, F***Rishetiakentungensis*, syntypes NHMUK 1903.7.1.3650 from Mong Sing, Siam Boundary **G***Rishetialimborgi*, holotype NHMUK 1903.7.1.3200 from Tenasserim **H, I***Rishetianathiana*, syntypes NHMUK 1906.1.1.2206 from Bassein District.

NHMUK 1903.7.1.3742 (7 shells + 1 broken shell; Fig. 14A, B) collected by F. Ede ex. Godwin-Austen collection from Somra Tracts, Somra Khulen Post, Burma (labelled as ‘var. somraensis’). NHMUK 1903.7.1.3744 (2 shells; Fig. 14C) collected by F. Ede ex. Godwin-Austen collection from Somra Tracts, Somra Khulen Post, Burma (labelled as ‘var. somraensis’). NHMUK 1988147 (4 shells; Fig. 13H) collected by L.R. Mawson from Somra Tracts, Somra Khulen Post, west of Kyengdwen River, Burma.

#### Diagnosis.

Shell elongate, turreted, and regularly attenuated; apex rounded and blunt; subsequent whorls nearly smooth with fine irregularly spaced radial ridges throughout, and coarser ridges appearing near suture. Suture impressed and whorls flattened. Aperture ovate; columella concave and truncated.

#### Distribution.

This subspecies was originally described from the Naga Hills. The locality reported in Myanmar is Somra Town (~ 25°21'43.6"N, 94°41'14.4"E), a mountainous area in Leshi or Layshi Township, Sagaing Region in northwestern Myanmar. It is neighbouring Nagaland to the west and Kachin State to the east and along the Chindwin River.

#### Remarks.

The name *maxwelli* was stated twice in the same publication, on pages 45 and 61. The first mention is ‘Glessula (Rishetia) burrailensis, var. maxwelli’; when proposing this name, Godwin-Austen (1920: 45) recognised two specimen lots from Naga Hills… (collected by Col. H. St. P. Maxwell) and from Somra… Upper Burma (collected by F. Ede). Although the species description is brief, Godwin-Austen provides the catalogue number ‘No. 1717 B.M.’, two sets of shell dimensions, and illustrations of two shells from Naga Hill. We have examined this specimen lot, NHMUK 1903.7.1.1717, from Naga Hills, which consists of four shells. In the original description, Godwin-Austen (1920: 46) further states, ‘Size: Fig. 5. Type’ and in the figure caption as ‘Fig. 5. (1^st^ Type) from Naga Hills’ and ‘Fig. 6. (2^nd^ Type) from Naga Hills’. It seemed that Godwin-Austen intended to designate the specimen figured in plate 160, fig. 5, as the unique name-bearing type, which we consider as the holotype. The other three specimens from the same lot are therefore paratypes.

The second mention is ‘*Glessulaburrailensis*, var. maxwelli’, for which Godwin-Austen (1920: 61) provided a very detailed description together with specimen lot number ‘No. 3742 B.M.’, two sets of shell measurements, from ‘Somra Tracts, Somra Khulen Post, Upper Burma, but without illustration. The NHMUK collections contain specimen lot NHMUK 1903.7.1.3742, consisting of eight specimens with the original label stating ‘var. somraensis, GA.’. This manuscript name has never properly been published and made available (ICZN 1999: Art. 10), the specimen lot NHMUK 1903.7.1.3742, with its original label stating ‘var. somraensis’ is considered as the syntype. Therefore, this second combination becomes an available name (ICZN 1999: Arts 11, 12), and it is considered a junior (primary) homonym (ICZN 1999: Arts 24.2.1, 24.2.2, 53.3, 57.2). Additionally, syntypes also include, NHMUK 1988147 (4 shells), from Somra, Khulen Post, West of Kyendwin River, Upper Burmah, as Godwin-Austen (1920: 61) recognised in the original description.

After examining the type specimens carefully, we found no differences in the diagnostic characters between the senior homonym (NHMUK 1903.7.1.1717/1 and NHMUK 1903.7.1.1717/2–4) and junior homonym (NHMUK 1903.7.1.3742, NHMUK 1903.7.1.3744 and NHMUK 1988147) specimens, especially shell shape, shell sculpture, and protoconch sculpture. The only detected difference is the brownish or yellowish brown periostracum, which we consider as intraspecific variation. Therefore, it seems unnecessary to propose a new replacement name for this junior homonym.

### ﻿﻿31 Rishetia hastula

Taxon classificationAnimaliaStylommatophoraSubulinidae

(Benson, 1860)

E2465C30-07A9-5AA4-A634-1E31C59E9105

Fig. 14D

Achatina hastula Benson, 1860: 461. Type locality: ad Pankabari, prope Darjiling [Pankhabari, near Darjiling, West of Bengal, India]. Pfeiffer 1868: 235.
Achatina (Electra) hastula —Hanley and Theobald 1870: 9, pl. 18, fig. 4.
Stenogyra (Glessula) hastula —Nevill 1878: 169.
Stenogyra (Subulina) hastula —Pfeiffer and Clessin 1881: 327.
Glessula hastula —Theobald and Stoliczka 1872: 334. Beddome 1906: 167. Pilsbry 1909: 93, pl. 12, fig. 12. Gude 1914: 414. Ramakrishna et al. 2010: 161
Glessula (Rishetia) hastul a—Godwin-Austen 1920: 16, 17, pl. 161. figs 16, 17, pl. 163. figs 9, 9a, 10.
Rishetia hastula —Budha et al. 2017: 139, figs 2d, 6. Preece et al. 2022: 127, 128, fig. 55a.

#### Type specimen.

***Neotype***NHMUK 1906.1.1.880 (designated in Preece et al. 2022: 127, fig. 55a) (Fig. 14D) ex. Blanford collection from Darjiling.

#### Diagnosis.

Shell elongate, turreted, and regularly attenuated; apex rounded and blunt; subsequent whorls coarse with fine equally spaced radial ridges throughout. Suture somewhat impressed and whorls convex. Aperture broadly ovate; columella strong, concave, and truncated.

#### Distribution.

The species was originally described from India and later recorded from Nepal (Ramakrishna et al. 2010; Budha et al. 2015; Kalita 2022). In Myanmar, it was reported from Kumah Hill and Maii [Ma-ei Town ~ 19°20'36.5"N, 94°08'21.9"E], which are in the Thandwe District, Rakhine State in the westernmost part of Myanmar (Theobald and Stoliczka 1872; Gude 1914; Godwin-Austen 1920).

#### Remarks.

To clarify the taxonomic status of this species, Preece et al. (2022) have designated a neotype based on the specimen figured in Hanley and Theobald (1870, pl. 18, fig. 4). However, the image used to represent the neotype in Preece et al. (2022: fig. 55a) is an erroneous repetition of ‘*Achatinaleptospira* Benson, 1865’ (see Preece et al. 2022: fig. 51f). Therefore, the correct image of the neotype NHMUK 1906.1.1.880 is illustrated herein.

Theobald and Stoliczka (1872: 334) noted that the specimens from Arakan tended to differ from the type specimen in having a larger shell size. Nevertheless, Arakan and Sikkim are non-adjacent regions and far apart, which raises doubts about whether they are the same or distinct species.

### ﻿﻿32 Rishetia kentungensis

Taxon classificationAnimaliaStylommatophoraSubulinidae

(Godwin-Austen, 1920) comb. nov.

76279DD8-E0D3-5E9C-9599-8602E9479B71

Fig. 14E, F

Glessula kentungensis Godwin-Austen, 1920: 57, 58. Type locality: Mong Sing, Siam Boundary [Muang Sing District, Luang Namtha Province, Laos]. Inkhavilay et al. 2019: 49, fig. 20b.

#### Type specimens.

***Syntypes***NHMUK 1903.7.1.3650 [re-registered in error as 1986002] (3 shells + 1 juvenile; Fig. 14E, F) collected by Woodthorpe ex. Godwin-Austen collection from Mong Sing, Siam Boundary.

#### Other material.

NHMUK 1903.7.1.3748 (4 shells) collected by Woodthorpe ex. Godwin-Austen collection from the Mekong River.

#### Diagnosis.

Shell elongate, turreted, and regularly attenuated; apex rounded, blunt and with very large embryonic shell; subsequent whorls nearly smooth throughout, and prominent irregularly spaced radial ridges near suture. Suture impressed and whorls slightly flattened. Aperture broadly ovate; columella strong, concave, and truncated.

#### Distribution.

This species was initially found in Laos; however, as noted by Godwin-Austen (1920), its presence in the Mekong River region is assumed to likely extend to the East Shan State.

#### Remarks.

The original description gave the measurements for only one shell and did not include an illustration. Godwin-Austen (1920) clearly states the collection locality and the catalogue number ‘Type No. 3650 B.M.’. The NHMUK collections contain a lot of four specimens from the Godwin-Austen ex. Woodthorpe collection with an original label stating ‘Type’ and giving the collection locality as ‘Mong Sing, Siam Boundary’. The two specimens match well with the original description, and shell dimensions are illustrated herein. Another specimen, lot ‘No. 3748 B.M.’ from the ‘Mekong River’, is excluded from the type series of this nominal species.

Originally, the type locality was said to be from ‘Mong Sing, Siam Boundary’; currently, this locality refers to Muang Sing District, Luang Namtha Province, Laos. The specimen NHMUK 1903.7.1.3748, collected from the ‘Mekong River’ by Colonel R. G. Woodthorpe, has a vague locality that could possibly encompass the East Shan State (formerly Keng Tung State) and the Bokeo and Luang Namtha provinces in Laos.

### ﻿﻿33 Rishetia limborgi

Taxon classificationAnimaliaStylommatophoraSubulinidae

(Godwin-Austen, 1920) comb. nov.

BA3286E5-3BB5-5905-A10F-D1B039031296

Fig. 14G

Glessula limborgi Godwin-Austen, 1920: 56, 57. Type locality: Tenasserim [Tanintharyi Region, Myanmar].

#### Type specimen.

***Holotype***NHMUK 1903.7.1.3200 [re-registered in error as 1985219] (Fig. 14G) collected by Limborg ex. Godwin-Austen collection from Tenasserim.

#### Diagnosis.

Shell elongate, turreted and regularly attenuated; apex rounded; subsequent whorls with fine and equally spaced radial ridges throughout. Suture impressed and whorls flattened. Aperture broadly ovate; columella strong, concave, and truncated.

#### Distribution.

This species is known only from the type locality and is likely endemic to that region.

#### Remarks.

Godwin-Austen (1920) clearly states that this taxon was described based on only a single specimen collected by O. Limborg. The original description included only a single set of shell measurements but was without illustration. The NHMUK collections contain the type specimen lot, NHMUK 1903.7.1.3200 ex. Godwin-Austen ex. O. Limborg consisting of a single shell with an original label stating ‘Type’. Therefore, we considered this shell as the holotype fixed by monotypy.

### ﻿﻿34 Rishetia nathiana

Taxon classificationAnimaliaStylommatophoraSubulinidae

(Godwin-Austen, 1920) comb. nov.

4A29A2B4-EA44-5C9E-BFF5-32E26F90D0D7

Fig. 14H, I

Glessula (Rishetia) nathiana Godwin-Austen, 1920: 54, 55. Type locality: Bassein District [Pathein District, Ayeyarwady Region, Myanmar].

#### Type specimens.

***Syntypes***NHMUK 1906.1.1.2206 [re-registered in error as 1986018] (5 shells; Fig. 14H, I) ex. Blanford collection from Bassein District.

#### Diagnosis.

Shell elongate turreted and regularly attenuated; apex rounded and blunt; subsequent whorls with very fine equally spaced radial ridges, which coarser near suture. Suture impressed and whorls flattened convex. Aperture broadly ovate; columella concave and truncated.

#### Distribution.

*Rishetianathiana* is known only from the type locality and is likely endemic to that region.

#### Remarks.

Godwin-Austen clearly stated that the original description was based on a specimen lot, ex. W.T. Blanford collection with the catalogue number ‘Type No. 2206.06.1.1 B.M.’. The NHMUK collections contain a lot of five specimens from the Godwin-Austen ex. Blanford collection with original label ‘Type’ and ‘ex. duplicate collection’. The specimen matches well with the given shell measurements, and the original description is illustrated herein for the first time.

### ﻿﻿35 Rishetia pertenuis pertenuis

Taxon classificationAnimaliaStylommatophoraSubulinidae

(Blanford, 1865) comb. nov.

FC8C1BBF-5612-5CE2-A97B-F3D1B1A13513

Fig. 15A–E

Achatina pertenuis Blanford, 1865: 79. Type locality: Tongoop, Arakan [Taungup Township, Rakhine State, Myanmar]. Pfeiffer 1868: 237.
Achatina (Electra) pertenuis —Hanley and Theobald 1870: 9, pl. 18, fig. 5.
Stenogyra (Glessula) pertenuis —Nevill 1878: 169.
Stenogyra (Subulina) pertenuis —Pfeiffer and Clessin 1881: 327.
Glessula pertenuis —Beddome 1906: 160. Gude 1914: 380. Godwin-Austen 1920: 52–54, pls 164, fig. 11, pl. 159, figs 1, 2. Ramakrishna et al. 2010: 170.
Glessula tenuispira var. pertenuis —Pilsbry 1909: 89, 90, pl. 9, fig. 3.

#### Type specimen.

**Figure 15.**
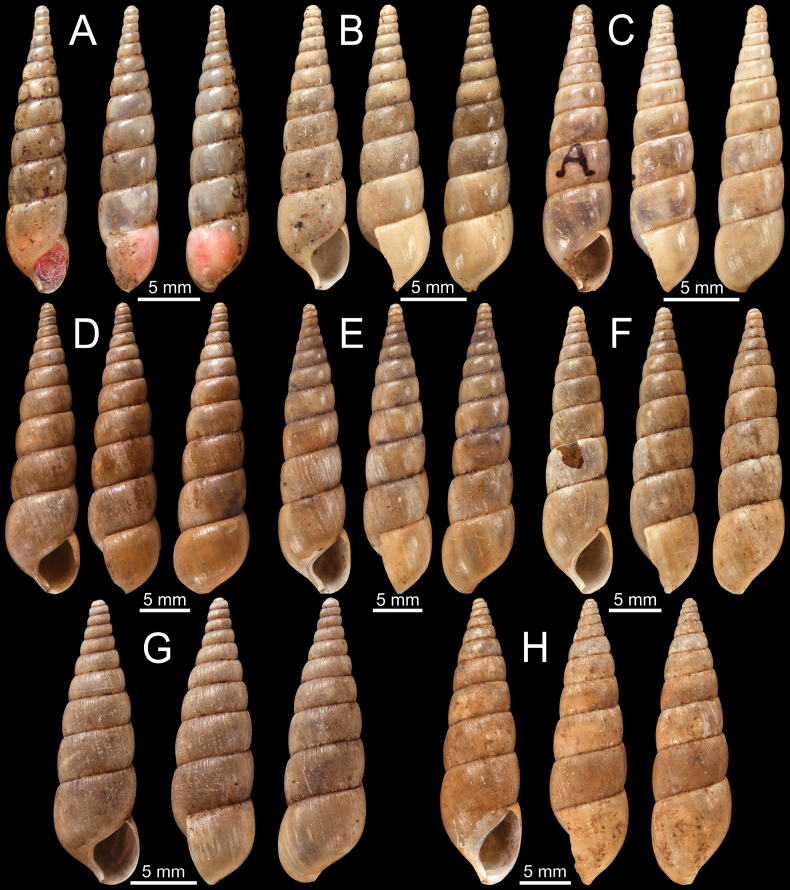
**A–E***Rishetiapertenuis***A, B** possible syntypes NHMUK 1906.2.2.239 from Henzada, Pegu and Tongoop, Arakan **C** specimen NHMUK from Tongoop, Arakan and **D, E** specimen NHMUK 1888.12.4.1229–31 from Pegu **F, G***Rishetiapertenuismajor*, possible syntypes NHMUK 1906.1.1.2208 from Pyema Khyoung, Bassein **H***Rishetiatenuispira*, specimen NHMUK 1903.7.1.3332 from Garo Hills.

***Possible syntypes***NHMUK 1906.2.2.239 (7 shells; Fig. 15A, B) ex. Blanford collection from Henzada, Pegu and Tongoop, Arakan.

#### Other material.

NHMUK 1888.12.4.1229–31 (3 shells; Fig. 15D, E) from Pegu. NHMUK 20230921 (5 shells) from Tongoop, Arakan (Fig. 15C).

#### Diagnosis.

Shell slender, elongate turreted, and rapidly attenuated near apical whorls; apex rounded; subsequent whorls nearly smooth, fine growth lines, and with equally spaced ridges near suture. Suture impressed and whorls slightly convex. Aperture ovate; columella concave and truncated.

#### Distribution.

This species was reported from several places in Myanmar: Henzada [Hinthada City, Ayeyarwady Region], Thayet Myo [Thayet City, Thayet District, Magway Region], Pegu [Bago Region], and Tongoop, Arakan [Taungup Township, Thandwe District, Rakhine State] (Nevill 1878; Gude 1914). It was also reported from Meghalaya State, India (Ramakrishna et al. 2010).

#### Remarks.

The original description does not clearly state how many specimens were available to the author, although only one set of measurements was given. The NHMUK collection contains a lot of seven specimens from the Blanford collection with this species name. However, this is a mixed specimen lot of the same species as it consists of two collection localities, ‘Henzada, Pegu’ and ‘Tongoop, Arakan’, where only the latter was mentioned in the original description as the type locality. Therefore, this specimen lot NHMUK 1906.2.2.239 is considered as possible syntypes, from which two specimens are illustrated herein.

### ﻿﻿36 Rishetia pertenuis major

Taxon classificationAnimaliaStylommatophoraSubulinidae

(Blanford, 1865) comb. nov.

48ADEA1E-AE46-5ABB-AEAC-F53C3D790051

Fig. 15F, G

Achatina pertenuis var. major Blanford, 1865: 79. Type locality: Pyema Khyoung, Bassein District, Pegu [PyayMa Khaung or Pyinma Khuang, Pathein Township, Pathein District, Ayeyarwady Region, Myanmar].
Glessula pertenuis var. major —Gude 1914: 381. Godwin-Austen 1920: 53.

#### Type specimen.

***Possible syntypes***NHMUK 1906.1.1.2208 (6 shells; Fig. 15F, G) ex. Blanford collection from Pyema Khyoung, Bassein; label state ‘*Glessulabasseinensis* G-A.’

#### Diagnosis.

Shell elongate, turreted and regularly attenuated; apex rounded and blunt; subsequent whorls with fine equally spaced radial ridges throughout, which coarser near suture. Suture impressed and whorls slightly flattened. Aperture broadly ovate; columella strong concave and truncated.

#### Distribution.

This subspecies was known solely from the type locality.

#### Remarks.

When describing ‘*Achatinapertenuis*’, Blanford (1865) recognised the larger shells as a distinct variety and gave them the name ‘var. major’. This taxon name was made available, although without description, because the two sets of shell dimensions and materials from ‘Pyema Khyoung, Bassein district, Pegu’ confer the indication (ICZN 1999: Arts 11.9 and 12).

The type series of the taxa could not be traced. Only one specimen lot from the Godwin-Austen collection belongs to W.T. Blanford’s original type series., i.e., NHMUK 1906.1.1.2208, with the original label in Godwin-Austen’s handwriting marked ‘ex. duplicate collection Blf., intermediate between *A.tenuispira* and *A.pertenuis*’, with a species name ‘*Glessulabasseinensis* G-A.’, and collection locality ‘Pyema Khyoung, Bassein’. Therefore, this specimen lot is here considered as possible syntype material, and the two shells closest to the shell measurements given in the original description are illustrated herein.

The modern name of ‘Pyema Khyoung, Bassein District, Pegu’ is the PyayMa Khaung or Pyinma Khuang (~ 18°38'51.7"N, 95°45'46.9"E), Pathein Township, Pathein District, Ayeyarwady Region, Myanmar.

### ﻿﻿37 Rishetia tenuispira

Taxon classificationAnimaliaStylommatophoraSubulinidae

(Benson, 1836)

25E041A0-058D-5F59-88B3-EC47AC6BD220

Fig. 15H

Achatina tenuispira Benson, 1836: 353. Type locality: N.E. Frontier of Bengal. Pfeiffer 1848: 262. Reeve 1849a: Achatina, pl. 16. Benson 1860: 464. Pfeiffer 1860: 310, 311 pl. 25, figs 6, 7. Blanford 1865: 95.
Subulina tenuispira —Adams and Adams 1855: 110.
Achatina (Subulina) tenuispira —Pfeiffer 1856: 169.
Achatina (Electra) tenuispira —Hanley and Theobald 1870: 17, pl. 36, fig. 8.
Stenogyra (Glessula) tenuispira —Nevill 1878: 169.
Stenogyra (Subulina) tenuispira —Pfeiffer and Clessin 1881: 327.
Glessula tenuispira —Beddome 1906: 160. Pilsbry 1909: 88, pl. 9, figs 1, 4. Gude 1914: 378. Godwin-Austen 1920: 31, 32.
Rishetia tenuispira —Raheem et al. 2014: 138, fig. 90c. Budha et al. 2017: fig. 14c. Preece et al. 2022: 128, fig. 55b.

#### Type specimen.

The type specimen could not be located in the UMZC collection (Raheem et al. 2014; Preece et al. 2022). However, Budha et al. (2017: fig. 14a) figured a possible syntype of this species based on specimens ex. Benson collection from ‘Teria Ghat’, this specimen lot was not considered to form part of the type series by Preece et al. (2022).

#### Other material.

NHMUK 1946.10.16.1–7 (7 shells) ex. R. McAndrew collection from Pegu. NHMUK 1903.7.1.3332 (1 shell; Fig. 15H) labelled as ‘var.’ ex. Godwin-Austen collection from Garo Hills. UMZC I.102045 from Teria Ghat (Budha et al. (2017: fig. 14a) recognised this specimen as a possible syntype).

#### Diagnosis.

Shell elongate, turreted, and regularly attenuated; apex rounded; subsequent whorls with fine equally spaced radial ridges throughout and more prominent near suture. Suture impressed and whorls slightly flattened. Aperture elongate ovate; columella concave and truncated.

#### Distribution.

This species is broadly distributed in Bangladesh, India, and Nepal (Budha et al. 2015; Preece et al. 2022). It was also reported from several localities in the southwest and eastern Myanmar: Ayeyarwady, Bago, and Tanintharyi regions (Benson 1860; Blanford 1865; Beddome 1906).

#### Remarks.

Benson (1860: 464) reported that Theobald found a variety of ‘*Achatinatenuispira*’ on the banks of Irrawaddy [Ayeyarwady River] and from Phie Than [probably in Tanintharyi Region]. Blanford (1865) recorded the presence of a small-sized species in Akoutoung [Akauk Taung, Ayeyarwady Region] and further south, while Beddome (1906) noted its occurrence in Pegu [Bago Region].

### ﻿﻿Genus Tortaxis

Taxon classificationAnimaliaStylommatophoraSubulinidae

Pilsbry, 1906

0E793DAE-8ED4-5288-AD5A-B4DB6E2D22FD

Tortaxis Pilsbry, 1906: 5, 6. Zilch. 1959: 347. Schileyko 1999: 534. Schileyko 2011: 10. Do and Do 2014: 455.

#### Type species.

*Achatinaerecta* Benson, 1842, by original designation.

#### Diagnosis.

Shell slender, cylindrically shaped; spire high, mostly turreted, and gradually attenuated; embryonic whorls smooth, and subsequent whorls with nearly smooth to strong radial ribs. Aperture vertical, narrow, and oblong; columella concave or straight and with spiral fold below; columellar margin with or without expansion near umbilicus. Umbilicus narrowly opened or closed.

#### Remarks.

*Tortaxis* can be distinguished from other subulinid taxa from Myanmar, namely *Allopeas*, *Opeas*, and *Bacillum*, by having a slender, cylindrical, and turreted spire, flatter whorls, smooth embryonic whorls, a large and rounded apex, and a distinct spiral fold on the columella (Table 2).

The genus is mainly distributed in Southeast Asia and southern China and comprises 14 extant species (Pilsbry 1906; Schileyko 1999; MolluscaBase 2023) and one Miocene-amber fossil from China (Yu et al. 2023). In Indochina, nine species have been reported from Vietnam, and only one species from Thailand (Panha 1998; Schileyko 2011; Do and Do 2014). In this study, we propose one new species which marks the first record of the genus in Myanmar.

### ﻿﻿38 Tortaxis cylindropsis

Taxon classificationAnimaliaStylommatophoraSubulinidae

Man & Panha sp. nov.

5B4725DA-C666-50F7-AF82-C2B520109CA0

https://zoobank.org/084E4BD5-300A-4391-AF52-A6A8F6ADD951

**Figure 16.**
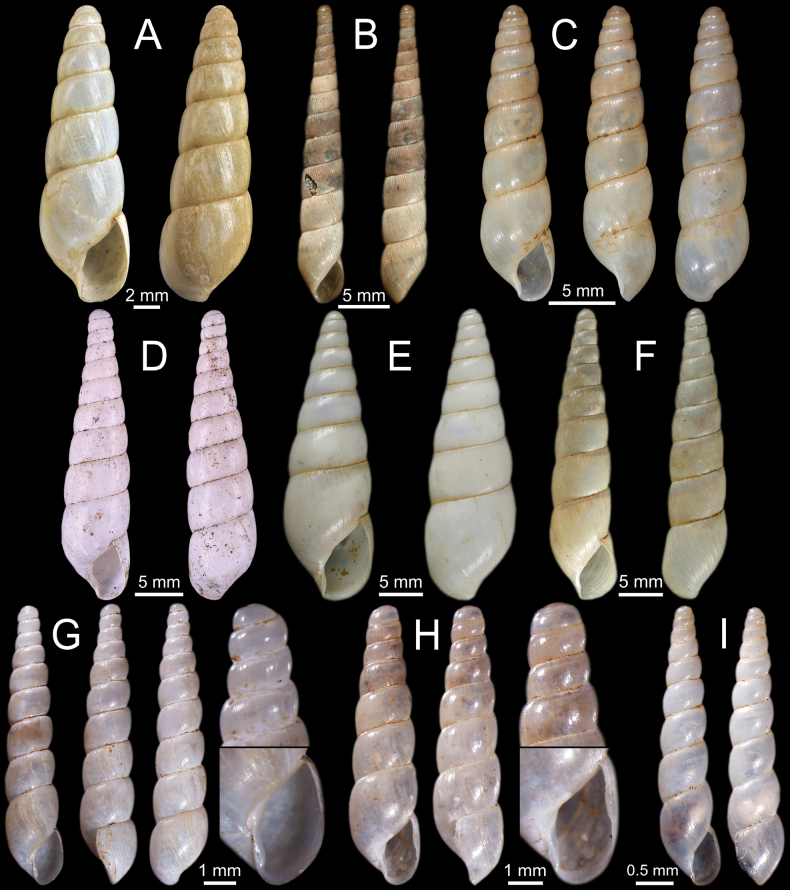
**A***Tortaxiserectus*, neotype NHMUK 1991104A from Nanking, China **B***Tortaxiselongatissimus*, syntype MNHN-IM-2000-4705 from Pac-Kha **C***Tortaxislubricus*, holotype ANSP 66056 from near Haiphong **D***Tortaxispapillosa*, MNHN-IM-2000-33971 from Nam-Nhang **E***Tortaxispermira*, syntype MNHN-IM-2000-4675 from That-Khé, Tonkin **F***Tortaxispilsbryi*, syntype MNHN-IM-2000-4678 from Bac-Khan et That-Khé **G–I***Tortaxiscylindrotis* sp. nov. **G** holotype CUMZ 13078 from Taunggyi, Shan State, Myanmar with embryonic whorls and aperture **H, I** paratypes CUMZ 13079 from the type locality **H** probably juvenile shell with 7 whorls and **I** adult shell. Photographs: P Bourguignon & D Brabant (**B, D–F**).

Fig. 16G–I , Table 1

#### Type specimens.

***Holotype***CUMZ 13079 (height 9.6 mm, width 2.3 mm; Fig. 16G), ***paratypes***CUMZ 13080 (35 shells; Fig. 16H, I), NHMUK 20230919 (2 shells) and SMF (2 shells).

#### Type locality.

Parpant area, Taunggyi City, Taunggyi District, Shan State, Myanmar (20°15'3.7"N, 97°14'23.9"E).

#### Etymology.

The specific name *cylindropsis* is from the Latin word for cylinder, and the Greek suffix -*opis* means ‘having the appearance of or like’. It refers to the cylindrical shell shape of this species.

#### Diagnosis.

Shell slender cylindrical, suture deeply impressed, spire distinctly turreted, protoconch rounded and smooth, subsequent whorls with fine growth lines and a distinct spiral fold on columellar margin.

#### Description.

Shell (height 9.3–11.7 mm) slender cylindrical in shape,, translucent, whitish to pale yellowish colour, and with 8–9½ whorls. Apex rounded; protoconch ~ 2 whorls, rounded and with smooth surface; subsequent whorls with fine growth lines more distinct on last whorl. Spire high, grows evenly and is largely turreted; whorls flatly convex; last whorls slightly larger than preceding whorls; suture narrow, deep, and weakly crenulated. Aperture vertical, narrowly ovate, and elongate; peristome thin; columella straight; columellar margin slightly expanded with distinct spiral fold. Umbilicus closed.

#### Distribution.

This species is known only from its type locality.

#### Differential diagnosis.

Comparing this new species with the Vietnamese species, *T.comaensis* Do, 2014 has a much larger and taller shell (height 56.1–66.4 mm), an attenuated spire, coarser sculptures, a broad aperture, and a thickened peristome, whereas this new species display a much smaller shell (height 9.3–11.7 mm), grows evenly and has a cylindrical spire, smooth shell surface, narrow aperture, and thin peristome (Do and Do 2014). *Tortaxiserectus* (Benson, 1842) has a less turreted shell, wide and flatly convex whorls, and a shallow suture (Fig. 16A), while *T.cylindropsis* sp. nov. has a distinctly turreted, slender shell with narrow and convex whorls, and a deep suture. *Tortaxiselongatissimus* Bavay & Dautzenberg, 1909 possesses a more elongated shell with stronger radial ribs and a more attenuated spire (Fig. 16B) than this new species. *Tortaxislubricus* Pilsbry, 1906 has a broad last whorl, attenuated spire, wider aperture, and shallow suture (Fig. 16C); in contrast, the shell of *T.cylindropsis* sp. nov. presents narrowly and even whorls, cylindrical, slender, and turreted spire, narrow aperture, and deep suture. *Tortaxispapillosa* Dautzenberg & Fischer, 1908 shows a broad shell with an attenuated spire, rounded embryonic whorls, flatter whorls, and a shallow suture (Fig. 16D), whereas *T.cylindropsis* sp. nov. has a slender shell, with cylindrical spire, convex whorls, and deeper suture. *Tortaxispermira* (Ancey in Bavay & Dautzenberg, 1904) and *T.pilsbryi* (Ancey in Bavay & Dautzenberg, 1904) have a wider shell, with a shallower suture, gradually attenuated spire, and an aperture wider than that of *T.cylindropsis* sp. nov. (Fig. 16E, F).

#### Remarks.

This genus is reported for the first time in Myanmar, and the present finding expands the distribution range of the genus from China, Vietnam, Laos, and Thailand to include Myanmar.

### ﻿﻿Genus Zootecus

Taxon classificationAnimaliaStylommatophoraSubulinidae

Westerlund, 1887

8CF20C90-4FC9-5BF2-AA87-C2E579930A3A

Zootecus Westerlund, 1887: 75. Kobelt 1902: 1022, 1032. von Martens 1895: 103. Pilsbry 1906: 104. Gude 1914: 366. Zilch 1959: 355. Schileyko 1999: 519, fig. 678. Neubert 2003: 154.
Obeliscella —Jousseaume, 1889: 359.
Chilogymnus —Jousseaume, 1894: 289.

#### Type species.

*Pupainsularis* Ehrenberg, 1831, subsequent designation by Kobelt (1902: 1022).

#### Diagnosis.

Shell pupiform; spire high, broad, cylindrical, and apex pointed; embryonic whorls smooth surface; subsequent whorls with irregularly dense, fine, coarse, or weak radial striations. Aperture oblique, broad, and oblong or rounded; columella straight. Penis long slender tube, and with slightly thickened wall and conical at base; vagina very much larger and muscularly thicker than male organ.

#### Remarks.

*Zootecus* can be distinctly distinguished from all subulinid taxa in Myanmar by its pupiform shell, broad spire, straight columella, thickened whitish peristome, and irregular coarse striations (Table 2).

The genus is distributed from the Cabo Verde Islands and the Sahara, extending eastwards to Arabia, Socotra Island, India, and Southeast Asia (Gude 1914; Solem 1966; Schileyko 1999; Neubert 2003). Currently, the genus consists of nine species, and two are known from Myanmar (Gude 1914; MolluscaBase 2023).

### ﻿﻿39 Zootecus insularis

Taxon classificationAnimaliaStylommatophoraSubulinidae

(Ehrenberg, 1831)

FEC53D7D-EFD3-5AFC-B104-43C2466CB413

Fig. 17A–C

Pupa insularis Ehrenberg, 1831: 13. Type locality: In insula Cameran, quae prope Maris rubri ostium australe inter Loheiam et Moccham iuxta Arabiae felicis littus sita est [Cameran Island, Red Sea between Loheia and Mocha, near coast of Arabia]. Pfeiffer 1848: 307.
Bulimus insularis —Pfeiffer 1853: 403. Hanley and Theobald 1870: 11, pl. 22, fig. 10.
Pupa (Cylindrus) insularis —Nevill 1877: 22.
Stenogyra insularis —von Martens 1895: 106, pl. 8, figs 5, 6.
Zootecus insularis —Pilsbry 1906: 106–108, pl. 26, figs 21–25, 29–33. Gude 1914: 367, 368. Schileyko 1999: 519, 520, fig. 678. Neubert 2003: 154, 155, figs 1, 2. Raheem et al. 2014: 118, fig. 74c.

#### Type specimen.

**Figure 17.**
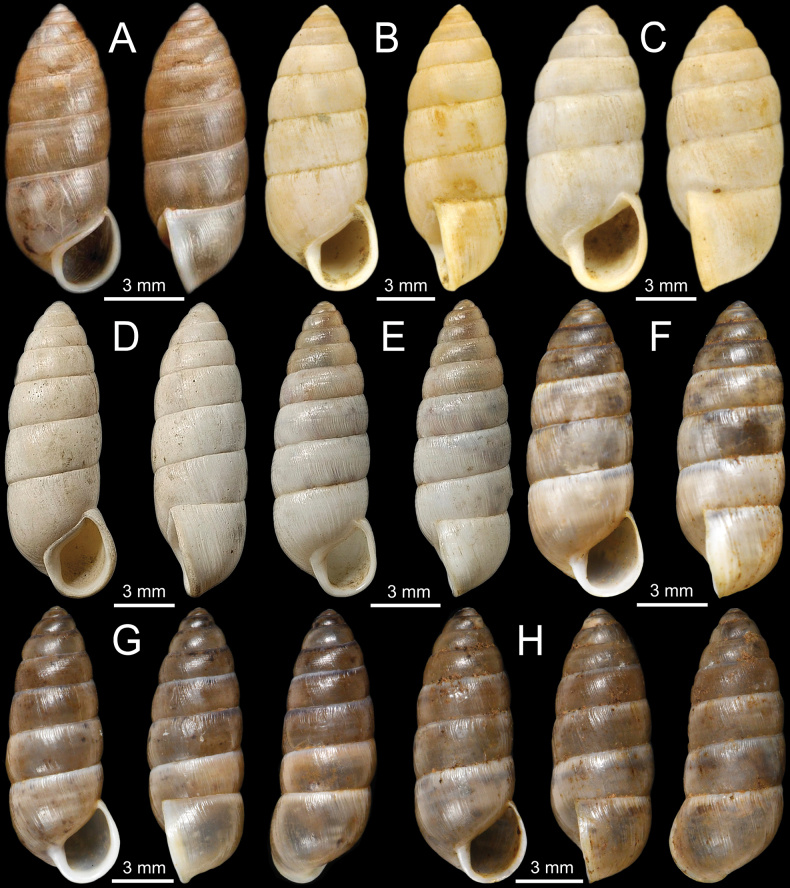
**A–C***Zootecusinsularis***A** lectotype ZMB 109990 (after Raheem et al. 2014: fig. 74c) and **B, C** specimen NHMUK 1875.12.4.16 from Burma **D–H***Zootecuspullus***D** lectotype NHMUK 1986252/1 and **E** paralectotype NHMUK 1986252/2 from Banks of the Ganges, South Asia and **F–H** specimen CUMZ 13081 from Bagan, Mandalay Region, Myanmar.

***Lectotype***ZMB 109990 (Fig. 17A, after Raheem et al. 2014: fig. 74c).

#### Other material.

NHMUK 1875.12.4.16 (5 shells; Fig. 17B, C) ex. Beddome collection from Burma. SMF 296651/2 (2 shells) ex. Ehrmann collection from Burma.

#### Diagnosis.

Shell subcylindrical; spire high and dome-shaped; apex pointed; subsequent whorls with fine and wavy radial striae, which stronger near suture. Aperture semi-ovate; columella short and straight. Umbilicus narrow.

#### Distribution.

*Zootecusinsularis* has a wide geographic distribution, spanning across the Cape Verde Islands, northeastern Africa, and the Arabian Peninsula. Its range also extends to South Asia, including India, Pakistan, Sri Lanka, and Afghanistan (Pilsbry 1906; Gude 1914; Neubert 2003; Raheem et al. 2014). In Myanmar, it was recorded from ‘Pagan’ [Bagan] in the Mandalay Region (Blanford 1865; Gude 1914).

#### Remarks.

No new specimens of this species were collected during this survey. However, the specimens collected from Bagan in the Mandalay Region are here identified as *Z.pullus* rather than *Z.insularis* (see under *Z.pullus* for further comparison). The historical museum specimens with brief locality records such as ‘Burma’ (Fig. 17B, C) match well with the respective type specimens of this species (Fig. 17A). As a result, the existence of *Z.insularis* in Myanmar requires further evidence from newly collected specimens with precise collection locality data, otherwise it must be excluded from the faunal list.

### ﻿﻿40 Zootecus pullus

Taxon classificationAnimaliaStylommatophoraSubulinidae

(Gray, 1834)

B4CAA9BC-F2E4-55F7-A1FD-D8DB3F812010

**Figure 18.**
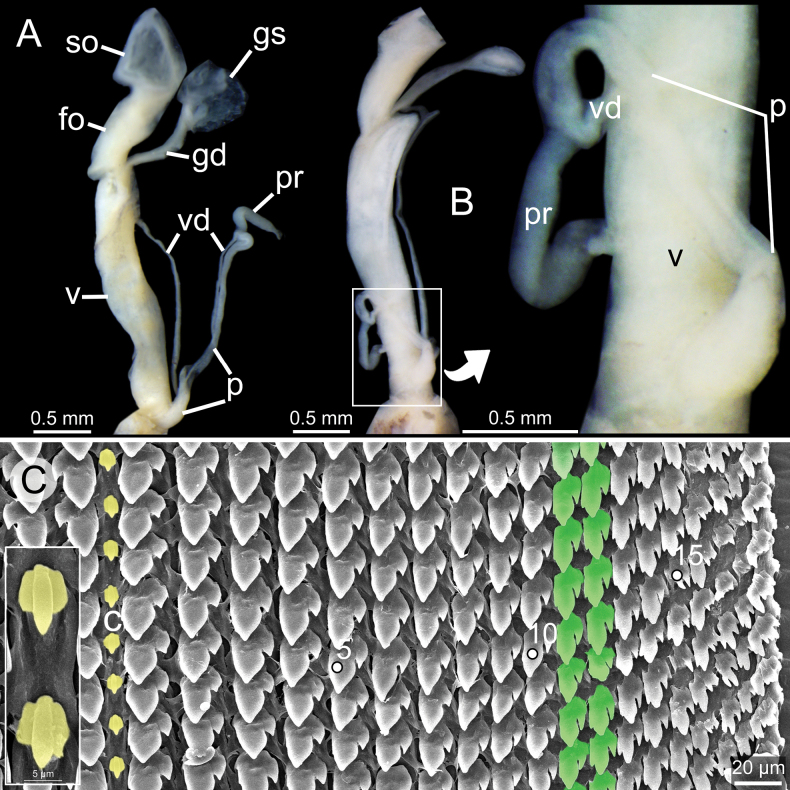
Genitalia and radula of *Zootecuspullus*, specimen CUMZ 13082 from Bagan, Mandalay Region, Myanmar **A, B** reproductive anatomy with inset of the male organ and **C** radula morphology with inset of central teeth: yellow colour and ‘C’ indicates central teeth row, green colour indicates lateral teeth in the transition to marginal teeth, and numbers indicate tooth order from lateral to marginal end. Abbreviations: **fo**, free oviduct; **gd**, gametolytic duct; **gs**, gametolytic sac; **p**, penis; **pr**, penial retractor muscle; **so**, spermoviduct; **v**, vagina; **vd**, vas deferens.

Figs 17D–H , 18 , Table 1

Bulimus pullus Gray, 1834: 66. Type locality: India Orientali ad ripas Gangis [Eastern India, banks of the Ganges River]. Pfeiffer 1848: 162. Reeve 1849b: Bulimus pl. 67, species 476. Blanford 1865: 94.
Bulimus (Opeas) pullus —Albers 1850: 175.
Pupa (Cylindrus) pulla —von Martens 1860: 297.
Bulimina (Mastus) pulla —Pfeiffer and Clessin 1881: 293.
Rumina pulla —Ancey 1886: 61.
Stenogyra pulla —von Martens 1895: 106, 107, pl. 8, figs 7, 8.
Zootecus insularis var. pullus —Pilsbry 1906: 110, pl. 26, figs 26–28.
Zootecus pullus —Gude 1914: 371, 372. Raheem et al. 2014: 118, 119, fig. 74e, f.

#### Type specimens.

***Lectotype***NHMUK 1986252/1 (Fig. 17D; designated in Raheem et al. 2014). ***Paralectotype***NHMUK 1986252 (1 shell; Fig. 17E) from Banks of the Ganges, South Asia.

#### Other material.

Dhammayazaka Pagoda, Pwasaw Village, Bagan City, Mandalay Region, Myanmar (21°08'40.3"N, 94°52'58.0"E): CUMZ 13081 (70 shells; Fig. 17F–H), CUMZ 13082 (20 specimens in ethanol).

#### Description.

Shell subcylindrical, solid, glossy, pale grey colour, slightly thick, and with 8–9½ whorls. Apex slightly elevated; protoconch ~ 2 whorls, dome-shaped and nearly smooth with fine radial striations; subsequent whorls with dense but fine, wavy, radial striations, stronger near suture. Spire grows evenly; whorls flatly convex; suture wide and shallow. Aperture nearly rounded and wide; columella straight; peristome relatively thickened, expanded, and white. Umbilicus narrowly opened.

***Genitalia*** (*n* = 5). Atrium undifferentiated. Penis very narrow, slender, almost same length with vagina, and slightly bulging at base. Penial retractor slender, long and attached at junction of penis and vas deferens. Epiphallus very short or indistinct. Vas deferens long, slender tube, and connected between penis/epiphallus to free oviduct (Fig. 18A, B).

Vagina much larger than penis and cylindrical shape. Gametolytic duct short and slender tube; gametolytic sac distinct and bulbus shape. Free oviduct almost the same diameter as vagina; spermoviduct enlarged.

***Radula*.** Each row contains ~ 43+ teeth with half-row formula: central-lateral-marginal teeth (1–(11–13)–(6–7+)). Central tooth relatively small, tricuspid with pointed central cusp, and small, rounded, lateral cusps. Lateral teeth bicuspid: endocone large rhomboid in shape and with pointed to dull tip; ectocone small, pointed tip and located at middle of tooth height. Marginal teeth asymmetrically tricuspid starting approximately at tooth number 11–13: mesocone large, triangular, and curved to blunt tip; endocone small and located near tip of mesocone; ectocone triangular, pointed tip and located near tooth base. Outermost teeth small and polycuspid (Fig. 18C).

#### Distribution.

Apart from Myanmar, this species is likely to occur in India and Bangladesh (Raheem et al. 2014).

#### Remarks.

In Myanmar, the previous records of *Z.pullus* were from ‘Ava’ [Innwa in Mandalay Region]. Our newly collected specimens from Bagan (~ 150 km southwest of Innwa) are identified as this species, but they present some differences such as having a long and narrower shell and a blunt apex. *Zootecuspullus* has a penis nearly equal in length to the vagina, and the penis is slightly enlarged with a conical shape at base near the atrium (Fig. 18A, B), while *Z.insularis* possesses a penis shorter than the vagina, and the evenly slender penis (see Schileyko 1999: fig. 678 from a near type locality in the Red Sea). Further investigation is required to determine whether these two Myanmar species are distinct or simply demonstrate variation in shell morphology.

## ﻿﻿Conclusions

This study elucidates the historically known species of the Subulinidae in Myanmar, identifying nine genera and 40 taxa, including 2 newly described species. Of these, 17 species are restricted only to Myanmar, while the remaining species are also found in India, China, and other Southeast Asian countries. The genera *Glessula* and *Rishetia* represent the highest number of species, 12 and 10 species, respectively, and they are predominantly found in Myanmar rather than other countries in Indochina. The globally distributed *Allopeasgracile* and the newly described species *Tortaxiscylindropsis* sp. nov. are presently documented as the only species of these genera in Myanmar.

Except for *Opeasinnocens*, all other unique name-bearing types of the subulinid species recorded from Myanmar have been examined, revised, and are illustrated herein. Our revised taxonomy is grounded in literature records; however, the classification of one particular taxa that has never been illustrated or for which there are no available types remains somewhat arbitrary. Additionally, in regards to the distribution range of certain species, for instance e.g., *G.crassilabris*, *G.orophila*, and *R.hastula*, which extend well beyond their original type locality in India, further data is needed to confirm their occurrence in Myanmar.

The distribution of these subulinid snails sheds light on their historical prevalence. According to the literature, they are primarily found in regions such as Bago, Ayeyarwady, Magway, Rakhine, and Shan rather than in Mon and Kayin states, which are known for their rich limestone outcrops (Gude 1914; Godwin-Austen 1920). Species belonging to the genera *Glessula*, *Opeas*, *Paropeas*, *Tortaxis*, and *Zootecus* were collected in Shan State, Mandalay, and Tanintharyi regions. However, our recent survey did not encounter representative taxa for *Bacillum*, *Curvella*, and *Rishetia*. Instead, species like *Paropeasturricula* and *Opeasfiliforme* have expanded their distribution from Thailand into Myanmar, and the genus *Tortaxis* is recorded for the first time. In contrast, other families like Ariophantidae (Pholyotha et al. 2020; Sutcharit and Pholyotha 2023), Clausiliidae (Man et al. 2023), Helicarionidae (Sutcharit et al. 2020a; Pholyotha et al. 2022a, b), Hypselostomatidae (Tongkerd et al. 2024), and Streptaxidae (Man et al. 2022) have yielded several species in Mon and Kayin states during these surveys. Hence, subsequent surveys should focus on regions such as Bago, Ayeyarwady, Magway, and Rakhine, aiming to confirm the identity of uncertain species and to undertake a thorough revision using freshly collected material. Moreover, the occurrence of new records suggests that additional taxa are likely to be discovered.

Budha et al. (2017) highlighted that shell sculptures may serve as valuable characters in distinguishing *Glessula* and *Rishetia*. Likewise, our limited new sample of *Glessula* demonstrates a range of shell surface variations from smooth to ribbed sculpture. Species like *G.mandalayensis* sp. nov. and *G.gemma* exhibit smooth to fine striations, while grooves are observed in *G.feddeni* and *G.latestriata*, and ribbed sculpture is characteristic of *G.blanfordiana*. Similarly, *Paropeas* is characterised by rough and compact radial striations, while *Allopeas* has finer striations compared to *Paropeas*. On the other hand, *Opeas* and *Tortaxis* display the very fine striations or smooth surfaces among these two genera. However, future research in subulinid taxonomy still requires the integration of genetic data, genital anatomy, and shell morphological traits for a more comprehensive understanding of their systematics.

## Supplementary Material

XML Treatment for Allopeas

XML Treatment for Allopeas gracile

XML Treatment for Bacillum

XML Treatment for Bacillum obtusum

XML Treatment for Bacillum theobaldi

XML Treatment for Curvella

XML Treatment for Curvella plicifera

XML Treatment for Curvella pusilla

XML Treatment for Curvella puta

XML Treatment for Curvella scrobiculata

XML Treatment for Glessula

XML Treatment for Glessula blanfordiana

XML Treatment for Glessula crassilabris

XML Treatment for Glessula feddeni

XML Treatment for Glessula gemma

XML Treatment for Glessula inedita

XML Treatment for Glessula latestriata

XML Treatment for Glessula mandalayensis

XML Treatment for Glessula orophila

XML Treatment for Glessula peguensis

XML Treatment for Glessula perlevis

XML Treatment for Glessula ponsiensis

XML Treatment for Glessula woodthorpei

XML Treatment for Glessula yuangensis

XML Treatment for Opeas

XML Treatment for Opeas filiforme

XML Treatment for Opeas innocens

XML Treatment for Paropeas

XML Treatment for Paropeas swettenhami

XML Treatment for Paropeas terebralis

XML Treatment for Paropeas turricula

XML Treatment for Paropeas walkeri

XML Treatment for Rishetia

XML Treatment for Rishetia akouktoungensis

XML Treatment for Rishetia baculina

XML Treatment for Rishetia basseinensis

XML Treatment for Rishetia burrailensis maxwelli

XML Treatment for Rishetia hastula

XML Treatment for Rishetia kentungensis

XML Treatment for Rishetia limborgi

XML Treatment for Rishetia nathiana

XML Treatment for Rishetia pertenuis pertenuis

XML Treatment for Rishetia pertenuis major

XML Treatment for Rishetia tenuispira

XML Treatment for Tortaxis

XML Treatment for Tortaxis cylindropsis

XML Treatment for Zootecus

XML Treatment for Zootecus insularis

XML Treatment for Zootecus pullus

## ﻿﻿Additional information

### Author ORCIDs

Nem Sian Man https://orcid.org/0000-0002-4453-734X

Jonathan D. Ablett https://orcid.org/0000-0002-7277-1934

Ngwe Lwin https://orcid.org/0000-0002-4394-3741

Chirasak Sutcharit https://orcid.org/0000-0001-7670-9540

Somsak Panha https://orcid.org/0000-0002-4431-2458

### Data availability

All of the data that support the findings of this study are available in the main text.
